# Circular RNA circNHSL1 promotes gastric cancer progression through the miR-1306-3p/SIX1/vimentin axis

**DOI:** 10.1186/s12943-019-1054-7

**Published:** 2019-08-22

**Authors:** Zhonglin Zhu, Zeyin Rong, Zai Luo, Zhilong Yu, Jing Zhang, Zhengjun Qiu, Chen Huang

**Affiliations:** Department of General Surgery, Shanghai General Hospital, Shanghai Jiaotong University School of Medicine, 650 Xinsongjiang Road, Songjiang District, Shanghai, 201600 China

**Keywords:** CircNHSL1, miR-1306-3p, SIX1, Vimentin, Metastasis, Gastric cancer

## Abstract

**Background:**

Mounting evidences indicate that circular RNAs (circRNAs) play vital roles in the development and progression of various cancers. However, the detail functions and underlying mechanisms of circRNAs in gastric cancer remain largely unknown.

**Methods:**

The expression profile of metastasis-related circRNAs was screened by RNA-seq analysis. qRT-PCR was used to determine the level and prognostic values of circNHSL1 in gastric cancer tissues. In vitro cell wound healing and transwell (migration and invasion) and in vivo tumorigenesis and metastasis assays were performed to evaluate the functions of circNHSL1. Luciferase reporter, RNA immunoprecipitation (RIP) and rescued assays were employed to confirm the interactions between circNHSL1, miR-1306-3p and SIX1. It’s widely accepted that as a mesenchymal marker, Vimentin promotes invasion and metastasis in various cancers. Luciferase reporter assay was used to determine the regulation of SIX1 on Vimentin. In addition, In situ hybridization (ISH) was performed to detect the level and prognostic values of miR-1306-3p.

**Results:**

We found that the level of circNHSL1 was significantly up-regulated in gastric cancer, and positively correlated with clinicopathological features and poor prognosis of patients with gastric cancer. Functionally, circNHSL1 promoted cell mobility and invasion, as well as in vivo tumorgenesis and metastasis. Mechanistically, circNHSL1 acted as a miR-1306-3p sponge to relieve the repressive effect of miR-1306-3p on its target SIX1. Moreover, SIX1 enhanced Vimentin expression in the transcriptional level through directly binding to the promoter domain of Vimentin, thereby promoting cell migration and invasion. In addition, miR-1306-3p was down-regulated and negatively correlated with pathological features and poor prognosis in gastric cancer.

**Conclusions:**

CircNHSL1 promotes gastric cancer progression through miR-1306-3p/SIX1/Vimentin axis, and may serve as a novel diagnostic marker and target for treatment of gastric cancer patients.

**Electronic supplementary material:**

The online version of this article (10.1186/s12943-019-1054-7) contains supplementary material, which is available to authorized users.

## Background

Gastric cancer is the fifth most common cancer and the third major cause of cancer-related deaths worldwide [1]. Direct infiltration, hematogenous metastasis, transcoelomic spread and lymphatic metastasis are the main pathways of metastasis for gastric cancer, which are the leading causes of poor prognosis for patients in the advanced stage. Despite of many advancements in diagnoses and treatments for gastric cancer, most patients are found to be in the advanced stage as soon as being diagnosed, and the overall 5-year survival rate is still less than 30% [2, 3]. Hence, it is extremely urgent to elucidate the molecular mechanisms underlying gastric cancer development and progression and discover novel molecular targets for early diagnoses and treatments.

Circular RNAs (circRNAs) have emerged as a new type of endogenous non-coding RNA [4, 5] and are characterized by a continuous covalently closed loop with a back splice site between 5′- and 3′-end, which is different from the formation of linear RNA [6–8]. The circular structure of circRNAs is responsible for their stable existence and high abundance in different species. Previous studies have proved their broadly evolutionary conservation, abundant presence in exosomes and plasma, tissue and space-time specificity [9, 10]. Thereby, circRNAs have great potential as diagnoses, treatments and prognosis biomarkers for various diseases, especially cancers. With the development of high-throughput sequencing technique and bioinformatics analysis, multiple circRNAs were discovered and confirmed to be involved in diversified biological processes, such as cell cycle, proliferation, apoptosis, autophagy, migration and invasion, in different types of cancers [11–15]. In recent years, circRNAs have been identified to play roles in several molecular mechanisms, such as sponging microRNAs (miRNAs), protein translation and binding to RNA-binding proteins [16, 17], of which miRNA sponge is the most common roles of circRNAs in the development and progression of tumors. Despite several circRNAs have been reported in gastric cancer, few tumor metastasis-related circRNAs and their functions and molecular mechanisms have been clearly elucidated.

As a member of the homeobox gene family, Sine oculis homeobox homolog 1 (SIX1) encodes a homeodomain-containing transcription factor [18, 19]. Ectopic expression of SIX1 ubiquitously exists in numerous types of cancer and plays important roles in both tumor initiation and progression [20, 21]. The overexpression of SIX1 correlates with advanced clinicopathological characteristics and poor prognosis in esophageal cancer, colorectal cancer and hepatocellular carcinoma [22–24]. It has been reported that SIX1 enhances TGFβ signaling and transcriptionally promotes VEGF-C expression, thereby promoting lymphangiogenesis and metastasis of cervical squamous carcinoma [25]. SIX1 could transcriptionally suppress the expression of miR-200 family and post-transcriptionally promote ZEB1 expression, consequently repressing E-cadherin expression and promoting epithelial-mesenchymal transition (EMT) in colorectal cancer [26]. In addition, SIX1 decreases the expression of p53 through a competitive mechanism involving simultaneous downregulation of ribosomal protein L26 (RPL26) and upregulation of miR-27a-3p in breast cancer [21]. However, little is known about the functions and regulatory mechanisms of SIX1 in gastric cancer. Vimentin is widely accepted as a mesenchymal biomarker to promote EMT of various cancer cells, thereby promoting invasion and metastasis in vitro and in vivo [27]. Bioinformatics analysis indicated that SIX1 shares the binding sites of the promoter domain of Vimentin.

In this study, we identified a novel metastasis-related circRNA circNHSL1 from exons of the NHS like-1 (NHSL1), with a circBase ID of hsa_circ_0006835. We found that circNHSL1 was up-regulated in both gastric cancer tissues and cell lines, and correlated with advanced clinical stage, distant metastasis, lymph node metastasis and poor prognosis. Importantly, circNHSL1 promoted invasion and metastasis of gastric cancer by acting as a miR-1306-3p sponge to relieve its repression on target SIX1. Furthermore, we firstly demonstrated that SIX1 enhanced the expression of Vimentin in transcriptional level by directly binding to the promoter domain of Vimentin. Collectively, our data show that circNHSL1 acts as an oncogenic gene in gastric cancer progression through miR-1306-3p/SIX1/Vimentin axis, and may serve as a novel diagnostic marker and target for treatment of gastric cancer patients.

## Methods

### Clinical specimens and ethical approval

There are two groups of gastric cancer and adjacent normal tissues were collected. The first group of 57 paired gastric cancer and matched normal samples from 2013 to 2014 was obtained from patients with primary gastric cancer during surgical resection in Shanghai General Hospital and immediately fixed in formalin. The group of samples was embedded in paraffin to construct tissue microarray (TMA), and the final TMA contained 54 paired gastric cancer samples. Another group of fresh frozen specimens (93 pairs) was collected from 2015 to 2017 and stored in liquid nitrogen. None of the patients received radiotherapy or chemotherapy before surgery. All clinicopathological diagnoses were confirmed by two pathologists according to the guidelines of the Union for International Cancer Control (UICC). The present study was approved by the Ethics Committee of Shanghai General Hospital. Written informed consent was obtained from all subjects before enrollment in this study.

### Cell lines and culture conditions

Human gastric cancer cell lines (MKN-28, AGS, MKN-45, BGC-823, MGC-803, HGC-27, SGC-7901) and normal human gastric epithelial cells-1 (GES-1) were obtained from Type Culture Collection of the Chinese Academy of Science (Shanghai, China). All cell lines were maintained in RPMI 1640 medium with 10% fetal bovine serum (FBS) (Gibco, Grand Island, NY, USA) and 1% penicillin-streptomycin at 37 °C in a humidified atmosphere containing 5% CO_2_.

### Transfection, oligonucleotides and plasmids

To regulate circNHSL1, miR-1306-3p and SIX1 expression, oligonucleotides and plasmids were constructed. The following siRNAs targeting circNHSL1 were designed by RiboBio (Guangzhou, China): si-circ-1 target, 5′-ACACAGCAAAGGATAAA-3′; si-circ-2 target, 5′-ACAGCAAAGGATAAAG-3′; and si-circ-3 target, 5′-CAAAGGATAAAGATGGAAA-3′. Full length circNHSL1 was cloned into the pEX-3 (GenePharma, Shanghai, China) overexpression vector. The mimics, inhibitor and negative controls for hsa-miR-1306-3p were purchased from RiBoBio (Guangzhou, China). The shRNA-SIX1 sequences were as follows: 5′-CCAGCTCAGAAGAGGAATT-3′ (target), 5′- CCAGCTCAGAAGAGGAATT-3′ (sense), and 5′-AATTCCTCTTCTGAGCTGG-3′ (antisense). The SIX1 gene was cloned into pLVX plasmids (HarO Life, Shanghai, China). The oligonucleotides and plasmids were transfected into cells with Lipofectamine™ 2000 (Invitrogen, Carlsbad, CA, USA) according to the manufacturer’s instructions.

### RNA extraction, nuclear-cytoplasmic fractionation, RNase R treatment and quantitative real-time PCR (qRT-PCR)

Total RNA was extracted from gastric cancer tissues and cell lines using TRIzol (TaKaRa, Shiga, Japan) according to the manufacturer’s instructions. Nuclear and cytoplasmic RNA fractionation was isolated with PARIS™ Kit (Invitrogen, USA) following the manufacturer’s instruction. For RNase R treatment, 10 μg total RNA was incubated for 15 min at 37 °C with 40 U RNase R (Epicentre Technologies, Madison, USA). For circRNA and mRNA, RNA was reversely transcribed into cDNA using a PrimeScript™ RT Master Mix reagent kit (TaKaRa, Shiga, Japan). For miRNA, cDNA was synthesized by the PrimeScript™ RT reagent kit (TaKaRa, Shiga, Japan). RNA expression was quantified by qRT-PCR with SYBR Premix Ex Taq™ (TaKaRa, Shiga, Japan). GAPDH or U6 were used as internal controls for circRNA, mRNA or miRNA. The 2^-ΔΔCt^ method was used to analyze relative expression levels. The specific primers were listed in Additional file 1: Table S1.

### Protein extraction and western blotting

Total protein was extracted from gastric cancer cells using radioimmunoprecipitation assay buffer with 1% protease inhibitor phenylmethanesulfony fluoride (Beyotime Biotechnology, Jiangsu, China). BCA protein assay kit (Beyotime Biotechnology, Jiangsu, China) was used to measure protein concentrations. SDS-polyacrylamide gel electrophoresis (SDS-PAGE) was performed with 30 μg of total protein for each sample. Then, the protein was transferred onto polyvinylidene fluoride (PVDF) membrane (Millipore, MA, USA). Next, the membrane was blocked in 5% non-fat milk at room temperature for 1.5 h, and then was incubated with primary antibody at 4 °C overnight. Next day, the secondary antibody was incubated at room temperature for 1 h. Each Protein band was visualized by ECL chemiluminescent reagent (Millipore, MA, USA). The antibodies were listed as follows: SIX1 (1:1000, Cell Signaling Technology, MA, USA); Vimentin (1:2000, Abcam, MA, USA); GAPDH (1:5000; Cell Signaling Technology, MA, USA).

### Immunohistochemical analysis (IHC)

After dewaxing and rinsing, the TMA was boiled in 10 mM sodium-citrate buffer for 5 min to retrieve antigen. Then, the TMA was blocked in 3% hydrogen peroxide for 10 min in case of the intervention of endogenous peroxidase activity. Thereafter, the TMA was incubated with the primary anti-SIX1 antibody (1:200, Cell Signaling Technology, MA, USA) at 4 °C overnight. Next day, the TMA was incubated with secondary antibody at room temperature for 30 min. Next, the TMA was stained with DAB and hematoxylin. Lastly, the TMA was covered with coverslips for microscopic observation.

### In situ hybridization (ISH)

The TMA was dealt with Proteinase K for 10 min at 37 °C after de-waxing and re-hydration, followed by incubating with hybridization mix for 1 h at 57 °C. Then, the TMA was blocked for 15 min in blocking solution and hybridized with digoxigenin (DIG)-labeled miR-1306-3p probes at 50 °C for 16 h. Next day, the TMA was treated with 0.5% blocking reagent for 30 min after washing twice. Then, the TMA was incubated with anti-DIG and horseradish peroxidase for 2 h at room temperature, followed by washing twice with TBST and dehydration with xylene. Finally, the TMA was covered with coverslips. The staining scores were assessed by two independent pathologists blinded to the clinicopathological data. The staining scores were based on two indicators: the staining intensity and the proportion of positively stained cells. The staining intensity was scored with the following score system, 0 (negative staining), 1 (weak staining), 2 (moderate staining) and 3 (strong staining).The proportion of positively stained cells was evaluated with five levels, 0 (< 10%), 1 (10–25%), 2 (25–50%), 3 (50–75%) and 4 (> 75%). The products of the above two indicators were considered the final score. The final scores were divided into two grades, low expression (≤4), high expression (> 4).

### Cell wound healing and transwell assays

For cell wound healing assay, the cells were firstly cultured to full confluence in 6-well plates. Subsequently, the cells were scratched with a 200 μl micropipette tip in the center of the well. Then, the cells were incubated with serum-free medium. Representative images were captured at 0 h and 24 h after injury. The width of wound healing was quantified and compared with baseline values. All experiments were repeated independently in triplicate.

For transwell assay, 2 × 10^4^ cells of each group in 200 μl serum-free medium were seeded in the upper chamber (8.0 μm pore, Corning, USA) without (migration) or with (invasion) Matrigel (BD Bioscience, USA). 600 μl RPIM medium with 10% FBS was added to the lower chamber. After incubating for 24 h, the upper chambers were fixed with 4% polymethanol for 30 min and then stained with 0.1% crystal violet for 30 min. The cells that migrated or invaded to the reverse side of the upper chambers were photographed. Five random fields were selected to calculate cells that migrated or invaded.

### Luciferase reporter assay

The luciferase reporter plasmids (pGL3-Firefly-Renilla containing circNHSL1 sequence and Mutant sequence, pGL3-Firefly-Renilla containing SIX1 3′-UTR sequence and Mutant sequence) were synthesized by GenePharma Co. (Shanghai, China). The firefly luciferase reporter plasmids (pGL4.27-Luc containing Vimentin promoter and Mutant sequence) and renilla luciferase plasmids (pRL) were generated by HarO Life Co. (Shanghai, China). The luciferase reporter plasmids were co-transfected into cells with mimics, inhibitor, LV-shSIX1 and pRL or pLVX-shSIX1 and pRL using Lipofectamine™ 2000 reagent. After 36 h, the firefly luciferase and renilla luciferase activity were detected. The effects of miR-1306-3p or SIX1 on luciferase reporter plasmids were calculated with the ratio of firefly luciferase/Renilla luciferase activity. All experiments were independently repeated in triplicate.

### RNA immunoprecipitation (RIP) assay

RIP assay was conducted with magna RIPTM RNA-binding Protein Immunoprecipitation kit (Millipore, Billerica, MA). MKN-28 cells were transfected with miR-1306-3p mimics or negative control. The cells were lysed in complete RNA lysis buffer after 48 h. Then, the RIP immunoprecipitation buffer including magnetic beads conjugated with negative control mouse IgG or human anti-AGO2 antibody (Mouse, Millipore, Billerica, USA) was added into cell lysates. Subsequently, the lysates were rotated overnight. Next day, after incubating with Proteinase K for 30 min, the immunoprecipitated RNA was extracted. Last, qRT-PCR and agarose gel electrophoresis were performed to identify the expression of circNHSL1 and miR-1306-3p.

### Animal experiments

To establish xenograft mouse models, the shRNA against circNHSL1 (the same target with si-circ-1) and negative control were cloned into pLL3.7 vector, and the full-length cDNA of circNHSL1 or a negative control were cloned into PLCDH-ciR vector, containing a front and back circular frame. Then, the stable cell lines with knockdown or overexpression of circNHSL1 were constructed with MKN-28 or SGC-7901 cells. For in vivo tumorigenesis assay, 1.0 × 10^7^ MKN-28 or SGC-7901 cells in 150 μl PBS were subcutaneously injected into left inguinal region of male BALB/c athymic nude mice (4 weeks old). Tumor volumes were calculated by the formula: tumor = (length×width^2^)/2 and measured every three days. Finally, the mice were sacrificed, and the volume and weight of tumors were detected.

For in vivo liver metastasis assay, 1.0 × 10^7^ cells were intravenously injected into ileocolic vein of nude mice as we did previously [28]. For in vivo peritoneal metastasis assays, 1.0 × 10^7^ cells were injected into the abdominal cavity of nude mice. After 4 weeks, the livers were removed and the peritoneal metastatic nodules were observed. The livers were paraffin-embedded and finally validated by hematoxylin and eosin (H&E) staining. The animal experiments were approved by the Institutional Animal Care and Use Committee of Shanghai General Hospital, and were performed according to the guidelines for the care and use of laboratory animals.

### Statistical analysis

The SPSS 22.0 software was conducted for statistical analyses. Data were calculated by the χ^2^ or Fisher’s exact test. The correlations were analyzed by Pearson’s test (r, P). Paired and unpaired continuous variables were compared by Student’s t-test or the Mann-Whitney U test. The survival curves were drawn using the Kaplan-Meier method and were analyzed by log-rank tests. *p* < 0.05 was considered statistically significant in all tests.

## Results

### CircNHSL1 is up-regulated in gastric cancer tissues and correlates with the progression and poor prognosis

**Fig. 1:**
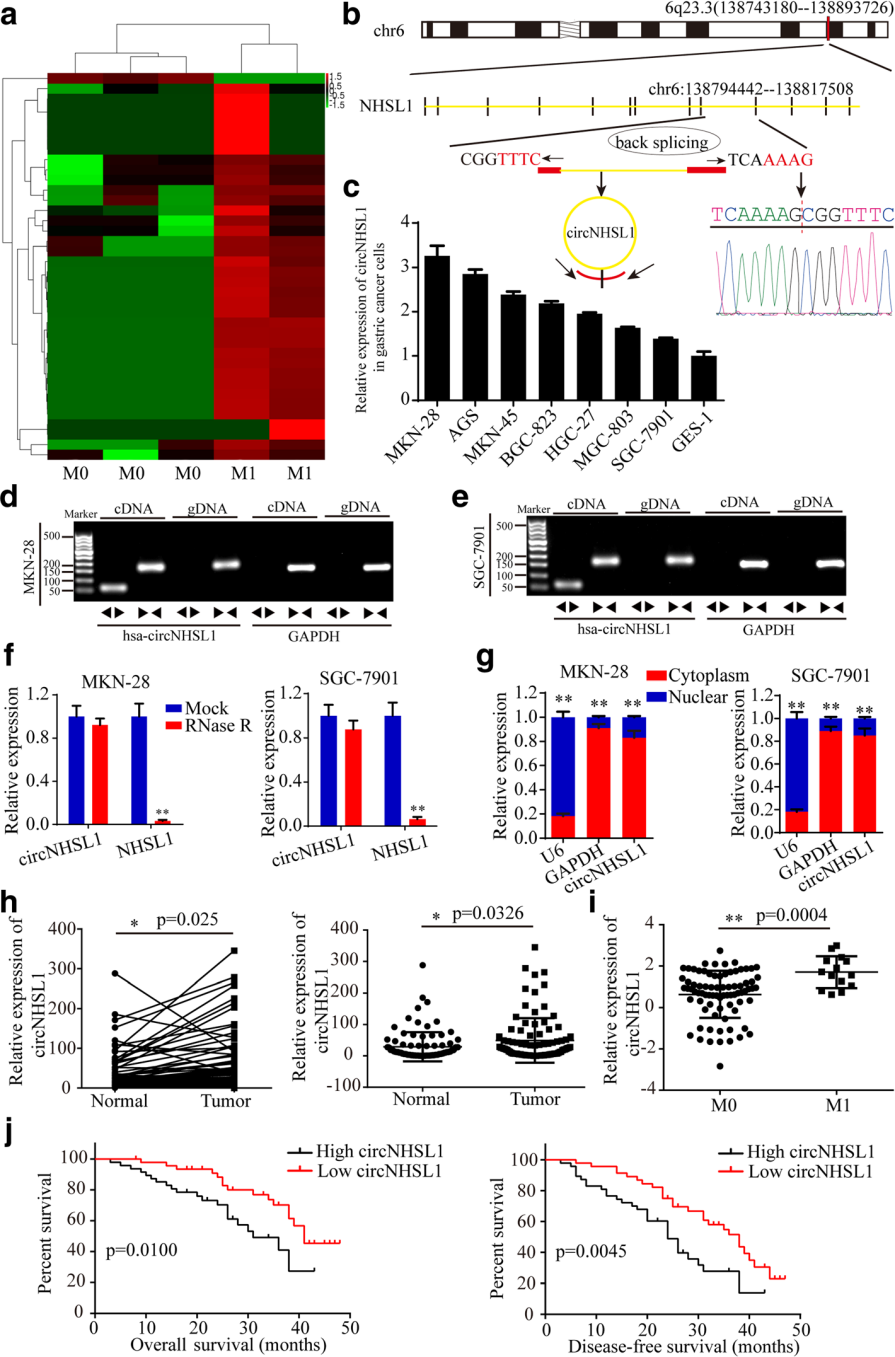
CircNHSL1 is up-regulated in gastric cancer tissues and correlated with the progression and poor prognosis. **a** CircRNA microarray between 3 gastric cancer tissues without metastasis and 2 gastric cancer tissues with metastasis. **b** Schematic illustration of the formation of circNHSL1 via the circularization of exons in NHSL1 gene. Sanger sequencing conformed the head-to-tail splicing of circNHSL1. **c** Relative expression of circNHSL1 in cell lines by qRT-PCR. **d** and **e** The gel electrophoresis validated the existence of circNHSL1. Divergent primers amplified circNHSL1 in cDNA but not gDNA. GAPDH was used as a linear control. **f** Relative expression of circNHSL1 and NHSL1 mRNA in both MKN-28 and SGC-7901 cells was detected by qRT-PCR in the presence or absence of RNase R. **g** circNHSL1 was mainly located in the cytoplasm by nuclear-cytoplasmic fractionation assay. **h** Relative circNHSL1 expression in 93 paired fresh frozen normal gastric tissues and gastric cancer tissues. **i** The level of circNHSL1 was significantly higher in gastric cancer tissues with M1 stage than with M0 stage (*p* < 0.01). **j** Kaplan-Meier survival analysis (log-rank test) showed that gastric cancer patients with high circNHSL1 expression have a lower OS and DFS than these with low circNHSL1 expression (*p* < 0.01). All data are presented as the mean ± SEM. *p < 0.05, **p < 0.01

To characterize metastasis-related circRNA transcripts, RNA-seq analysis between 3 gastric cancer tissues with metastasis and 2 gastric cancer tissues without metastasis was conducted (Fig. 1a). We found a total of 38 differentially expressed circRNAs with a cut-off criteria of fold change > 2.0 and p < 0.05, of which 37 were up-regulated and 1 was down-regulated in gastric cancer tissues with metastasis. In the top 10 up-regulated circRNAs, we found that circNHSL1, also named hsa_circ_0006835 according to the annotation of circBase (http://www.circbase.org/), was the highest up-regulated circRNA in gastric cancer tissues with metastasis, which was spliced from NHSL1 gene located at chr6: 138,743,180–138,893,726 and finally formed a sense-overlapping circular transcript of 335 nt (Fig. 1b). Sanger sequencing confirmed the head-to-tail splicing (Fig. 1b). To detect the level of circNHSL1 and NHSL1, we designed two sets of primers, divergent primers that were expected to amplify circNHSL1 and convergent primers to amplify linear NHSL1 mRNA. We found that the level of circNHSL1 was obviously higher in multiple gastric cancer cells than in GES-1 cell (Fig. 1c). Among these cell lines, MKN-28 and AGS cells exhibited the highest level of circNHSL1, while SGC-7901 and MGC-803 cells the lowest level (Fig. 1c). To detect whether the head-to-tail splicing of circNHSL1 results from trans-splicing or genomic rearrangements, we extracted cDNA and gDNA from MKN-28 and SGC-7901 cells. The gel electrophoresis results showed that circNHSL1 was detected only in cDNA, but not in gDNA, (Fig. 1d and e) indicating that the loop structure of circNHSL1 comes from reverse splicing. To confirm the stability of circNHSL1, MKN-28 and SGC-7901 cells were treated with RNase R, a processive 3′ to 5′ exoribonuclease. As shown in Fig. 1f, circNHSL1 resisted the digestion of RNase R, but the linear form of NHSL1 was digested sharply. In addition, the results of Nuclear-cytoplasmic fractionation illustrated that circNHSL1 was predominantly localized in the cytoplasm (Fig. 1g). These results indicate that circNHSL1 is highly stable in cytoplasm of gastric cancer cells, implying its potential to be an appropriate diagnostic or prognostic marker.

To determine the level of circNHSL1, we collected 93 pairs of fresh frozen gastric cancer tissues and matched normal tissues. qRT-PCR results showed that consistent with the results of gastric cancer cells, circNHSL1 expression was higher in most (80.65%, 75/93) gastric cancer tissues than in normal gastric tissues (Additional file 2: Figure S1, Fig. 1h). To further analyze the correlation between the level of circNHSL1 with clinicopathological features and prognosis, these samples were divided into two groups, high circNHSL1 group and low circNHSL1 group based on the median expression of circNHSL1. As shown in Table 1, high level of circNHSL1 was positively correlated with UICC stages, pathological T stages, lymphatic metastasis, distant metastasis and grades. Also, the level of circNHSL1 was higher in tissues with M1 stage than these with M0 stage (Fig. 1i), which confirmed the RNA-seq results that circNHSL1 is an oncogenic and metastasis-related circRNA. Furthermore, the Kaplan-Meier analysis showed that gastric cancer patients with high circNHSL1 expression had the significantly poorer OS and DFS compared with patients with low circNHSL1 expression (Fig. 1j). In summary, circNHSL1 was confirmed to be a highly stable circRNA and an appropriate diagnostic and prognostic marker for gastric cancer.

**Table 1:** Correlation between circNHSL1 expression and clinicopathological parameters in gastric cancer (*n* = 93)

Parameters	Category	No.	CircNHSL1 expression		*χ* ^*2*^	p
			Low (46)	High (47)		
Age					0.592	0.442
	< 65	57	30	27		
	≥65	36	16	20		
Gender					0.872	0.350
	Male	57	26	31		
	Female	36	20	16		
Differentiation					9.451	0.002
	Well	14	12	2		
	Mederate+ Poor	79	34	45		
T stage					6.412	0.011
	T1 + T2	29	20	9		
	T3 + T4	64	26	38		
N stage					4.111	0.043
	N0+ N1	33	21	12		
	N2+ N3	60	25	35		
M stage					5.470	0.019
	M0	79	43	36		
	M1	14	3	11		
UICC stage					9.451	0.002
	I	14	12	2		
	II + III	79	34	45		
Nerve invasion					0.093	0.761
	Yes	50	24	26		
	No	43	22	21		
Vessel invasion					0.104	0.747
	Yes	51	26	25		
	No	42	20	22		

### CircNHSL1 promotes migration and invasion of gastric cancer cells in vitro

**Fig. 2:**
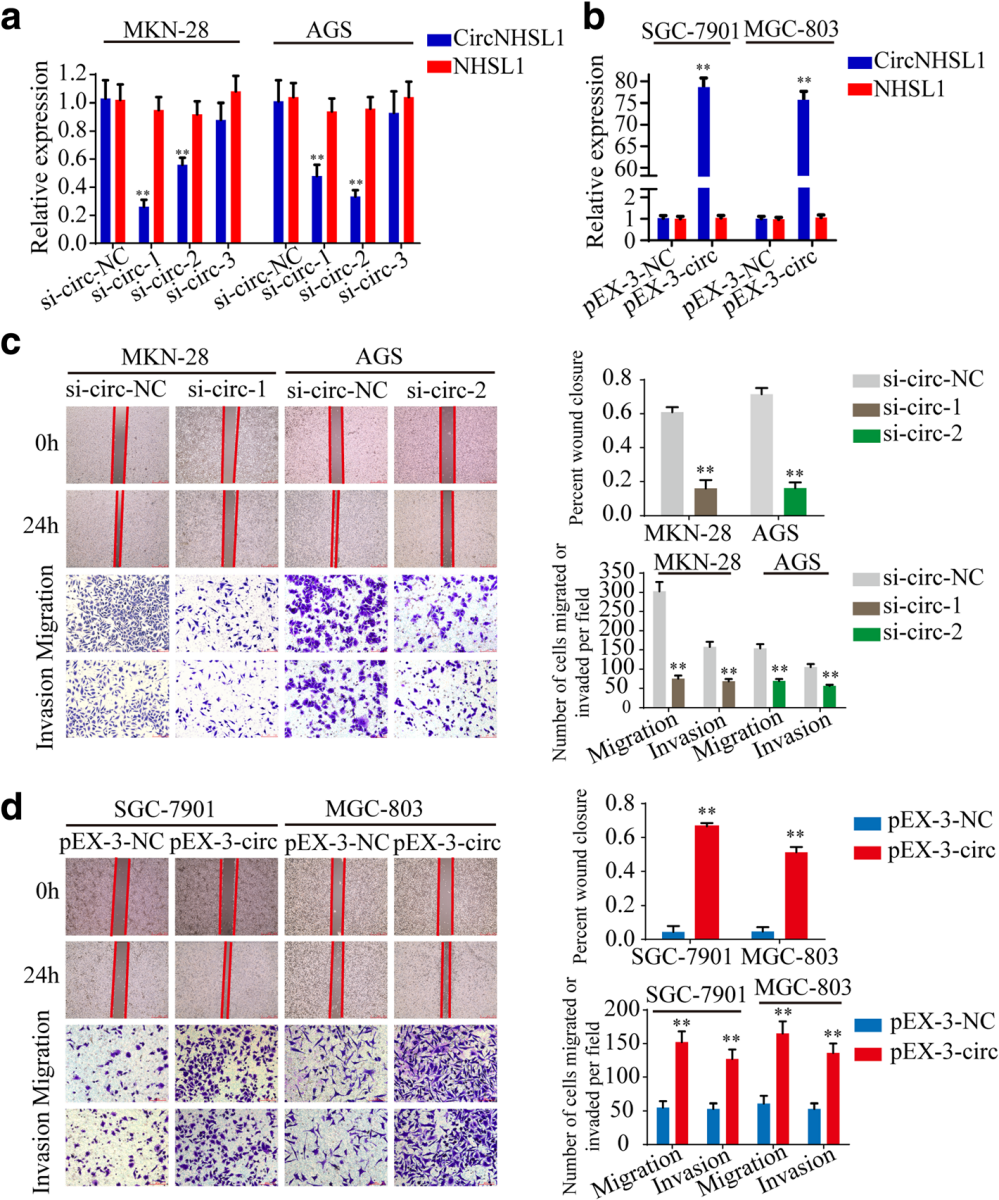
CircNHSL1 promotes migration and invasion of gastric cancer cells in vitro. **a** and **b** Relative expression of circNHSL1 and NHSL1 mRNA was detected by qRT-PCR in gastric cancer cells after transfection of si-circNHSL1, pEX-3-circNHSL1 or negative control. **c** The cell mobility, migration and invasion were evaluated by wound healing and transwell migration and invasion assays after knockdown of circNHSL1 in MKN-28 and AGS cells. **d** The cell mobility, migration and invasion were evaluated by wound healing and transwell assays after overexpression of circNHSL1 in SGC-7901 and MGC-803 cells. All data are presented as the mean ± SEM of three experiments. **p < 0.01

To test the functions of circNHSL1 in gastric cancer cells, three si-RNAs targeted the junction sites of circNHSL1 and overexpression plasmids of circNHSL1 were designed and transfected into MKN-28 and AGS cells or SGC-7901 and MGC-803 cells, respectively. CircNHSL1 expression was significantly silenced by si-circ-1 and si-circ-2, while NHSL1 mRNA did not change (Fig. 2a, Additional file 3: Figure S2b). Similarly, circNHSL1 was obviously overexpressed, and no significant change in NHSL1 mRNA was observed (Fig. 2b, Additional file 3: Figure S2a). Among the 3 si-RNAs, si-circ-1 had the highest knockdown efficiency in MKN-28 and SGC-7901 cells, while si-circ-2 in AGS cells. The mobility of gastric cancer cells was prominently decreased by down-regulation of circNHSL1, and the effect was also confirmed by the transwell migration and invasion assays in MKN-28, AGS and SGC-7901 cells (Fig. 2c, Additional file 3: Figure S2e and f). Nevertheless, overexpression of circNHSL1 promoted the mobility, migration and invasion of gastric cancer cells in SGC-7901, MGC-803 and MKN-28 cells (Fig. 2d, Additional file 3: Figure S2c and d). Taken together, these findings suggest that circNHSL1 promotes the progression of gastric cancer in vitro.

### CircNHSL1 promotes the progression of gastric cancer via SIX1

**Fig. 3:**
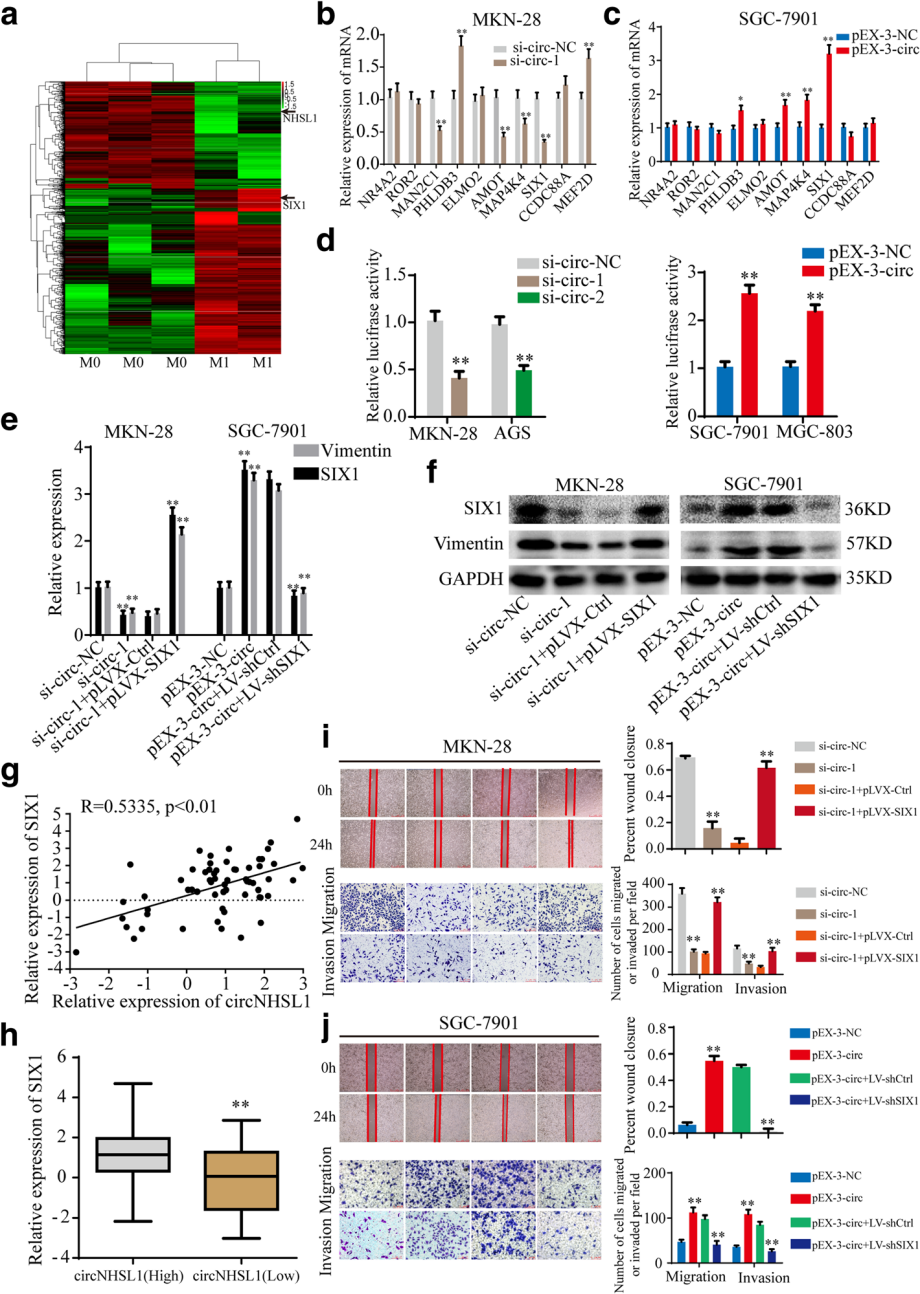
CircNHSL1 promotes the progression of gastric cancer via SIX1. **a** CircRNA microarray between 3 gastric cancer tissues without metastasis and 2 gastric cancer tissues with metastasis. **b** and **c** Relative expression of 10 mRNA candidates was detected by qRT-PCR in gastric cancer cells after transfection of si-circNHSL1, pEX-3-circNHSL1 or negative control. **d** Relative luciferase activities were detected in gastric cancer cells after transfecting luciferase reporter plasmid with wild type of SIX1 mRNA 3′-UTR with si-circNHSL1, pEX-3-circNHSL1 or negative control. **e** and **f** The effects of cicrNHSL1 and SIX1 on the mRNA and protein expressions of SIX1 and Vimentin were detected by qRT-PCR and western blotting. **g** Pearson correlation analysis determined the significantly positive correlation between the levels of circNHSL1 and SIX1 in 61 paired gastric cancer tissues (*p* < 0.01). **h** The level of SIX1 was higher in tissues with high circNHSL1 expression than with low circNHSL1 expression (p < 0.01). **i** and **j** The effects of cicrNHSL1 and SIX1 on the mobility, migration and invasion were confirmed by wound healing and transwell assays. All data are presented as the mean ± SEM of three experiments. **p < 0.01

According to the hypothesis of competing endogenous RNA (ceRNA) [29, 30], circRNAs may promote the expression of target genes by sponging miRNAs. Since circNHSL1 is located in the cytoplasm and exhibits high stability, we speculated that circNHSL1 could act as a miRNA sponge to increase target gene expression. To discover the genes positively correlated with circNHSL1 expression, RNA-seq for cDNA between above 3 gastric cancer tissues without metastasis and 2 gastric cancer tissues with metastasis in RNA-seq for circRNA was performed (Fig. 3a). The level of NHSL1 mRNA was low in gastric cancer tissues with metastasis. Combined with the results that NHSL1 expression was not changed by down-regulation or up-regulation of circNHSL1 in gastric cancer cells (Fig. 2a and b), circNHSL1 did not regulate NHSL1 mRNA expression through sponging miRNAs. The top 10 up-regulated genes in gastric cancer tissues with metastasis were selected. CircNHSL1 silencing significantly decreased MAP4K4 and SIX1 expression in both MKN-28 and AGS cells (Fig. 3b, Additional file 4: Figure S3a), while only AMOT and SIX1 expression increased in both SGC-7901 and MGC-803 cells after up-regulation of circNHSL1 (Fig. 3c, Additional file 4: Figure S3b). Thereby, we assumed that circNHSL1 positively regulated SIX1 expression. To confirm the hypothesis, a luciferase reporter plasmid with wild type of SIX1 mRNA 3′-UTR was constructed, and luciferase reporter assays were performed. CircNHSL1 silencing significantly decreased the luciferase activity, while circNHSL1 overexpression obviously increased the luciferase activity (Fig. 3d). Furthermore, down-regulation or up-regulation of circNHSL1 decreased or increased SIX1 protein expression in gastric cancer cells, respectively (Fig. 3e and f, Additional file 4: Figure S3c and d). We analyzed the level of circNHSL1 and SIX1 in 61 paired gastric cancer tissues among above 93 paired gastric cancer tissues, and found that the level of circNHSL1 positively correlated with the level of SIX1 (*p* < 0.01) (Fig. 3g). Similarly, the level of SIX1 in the high circNHSL1 group was significantly higher than in the low circNHSL1 group (Fig. 3h).

To test the effects of SIX1 on the functions of circNHSL1, cell wound healing, transwell migration and invasion assays were performed. The results showed that overexpression of SIX1 reversed the ability of circNHSL1 silencing to suppress the mobility, migration and invasion of gastric cancer cells (Fig. 3i), while down-regulation of SIX1 decreased the enhanced ability of mobility, migration and invasion induced by overexpression of circNHSL1 (Fig. 3j). In addition, circNHSL1 promoted the expression of mRNA and protein of Vimentin, while overexpression of SIX1 relieved the suppression of circNHSL1 silencing on Vimentin expression, and decrease in SIX1 attenuated the promotion of circNHSL1 overexpression on Vimentin expression, indicating that circNHSL1 promoted Vimentin expression via SIX1 (Fig. 3e and f). Taken together, circNHSL1 promotes gastric cancer progression via SIX1.

### SIX1 promotes the progression of gastric cancer by transcriptionally regulating vimentin expression

**Fig. 4:**
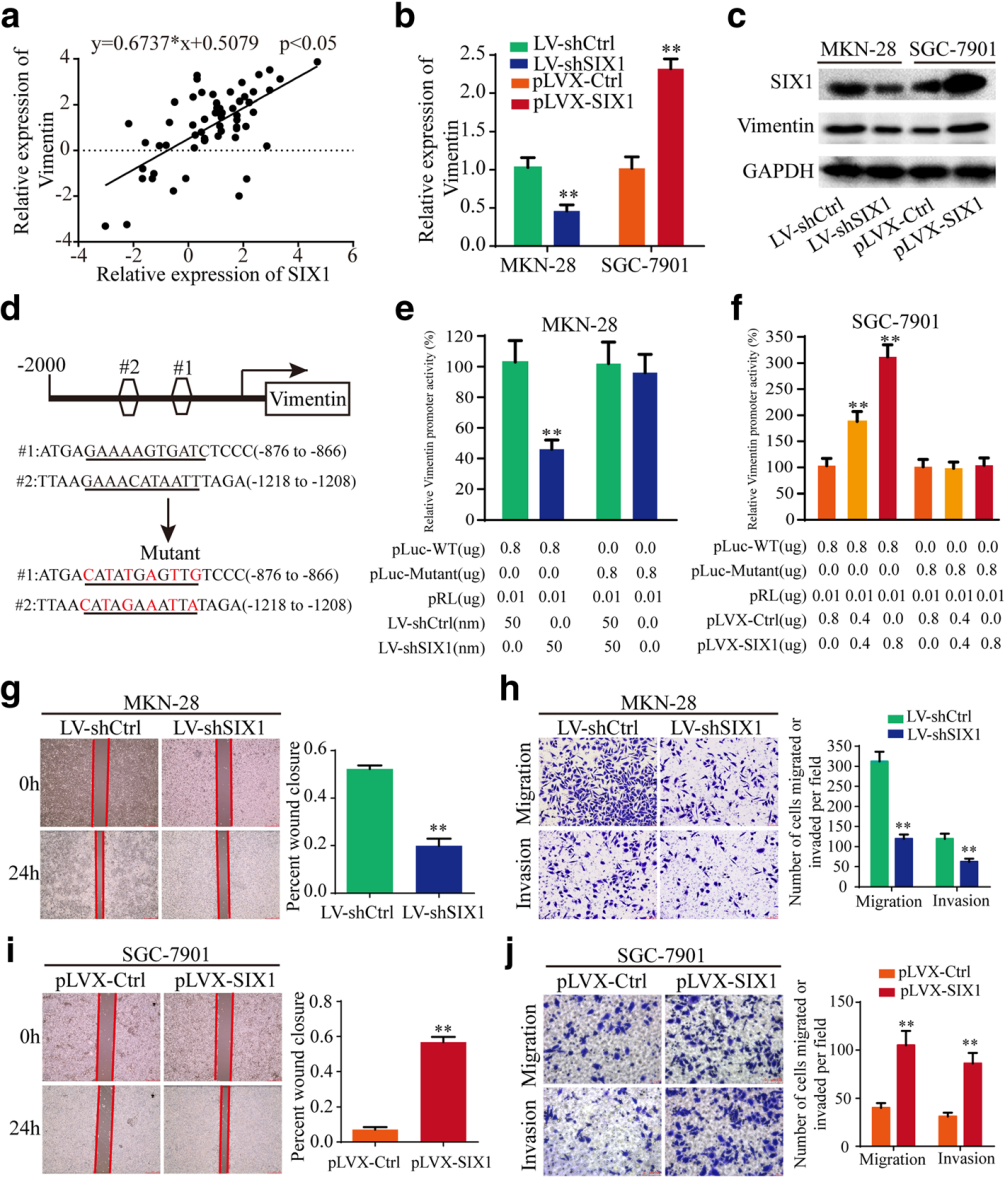
SIX1 promotes the progression of gastric cancer by transcriptionally regulating Vimentin expression. **a** The level of SIX1 positively correlated with the level of Vimentin in 61 paired gastric cancer tissues by Pearson correlation analysis (*p* < 0.01). **b** and **c** Relative expression of SIX1 and Vimentin was detected by qRT-PCR and western blotting in gastric cancer cells after transfection of LV-shSIX1, pLVX-SIX1 or negative control. **d** Schematic illustration of the sequences of wild type of Vimentin promoter domain and mutant sequences in the binding sites of SIX1 on Vimentin promoter domain are shown. **e** and **f** Relative luciferase activities were detected in gastric cancer cells after transfecting luciferase reporter plasmids with wild type of Vimentin promoter domain or mutant Vimentin promoter domain with LV-shSIX1, pLVX-SIX1 or negative control. **g-j** The effects of SIX1 on the mobility, migration and invasion were detected by wound healing and transwell assays. All data are presented as the mean ± SEM of three experiments. **p < 0.01

As a transcription factor of the homeobox gene family, SIX1 may regulate target gene expression in transcriptional level, thereby exerting biological functions. We analyzed the potential binding DNA sequence logo of SIX1 (Additional file 5: Figure S4a) and found two theoretical binding sites in the top 2000 nt of the promoter domain of Vimentin gene (http://jaspar.genereg.net) (Fig. 4d). Hence, we speculated that SIX1 may regulate Vimentin expression in the transcriptional level. Pearson correlation analysis showed that the mRNA expression level of SIX1 was positively correlated with that of Vimentin in above 61 paired gastric cancer tissues (*p* < 0.05) (Fig. 4a). SIX1 promoted Vimentin expression in the level of mRNA and protein, as shown in Fig. 4b and c. Furthermore, a luciferase plasmid with the top 2000 nt of the promoter domain of Vimentin gene (pLuc-WT) and a luciferase plasmid with mutant sequences in both two binding sites of the top 2000 nt of the promoter domain (pLuc-Mutant) were generated (Fig. 4d). Luciferase reporter assays demonstrated that SIX1 enhanced the luciferase activity of pLuc-WT in a dose-dependent manner, but not that of pLuc-Mutant (Fig. 4e and f), suggesting that SIX1 enhanced Vimentin expression by directly binding to the promoter domain of Vimentin. Then, we tested the functions of SIX1 in gastric cancer cells. The results illustrated that SIX1 promoted cell mobility, migration and invasion of gastric cancer cells (Fig. 4g-j). To summary, SIX1 promotes gastric cancer progression by positively regulating Vimentin expression in the transcriptional level.

### CircNHSL1 acts as a miRNA sponge of miR-1306-3p

**Fig. 5:**
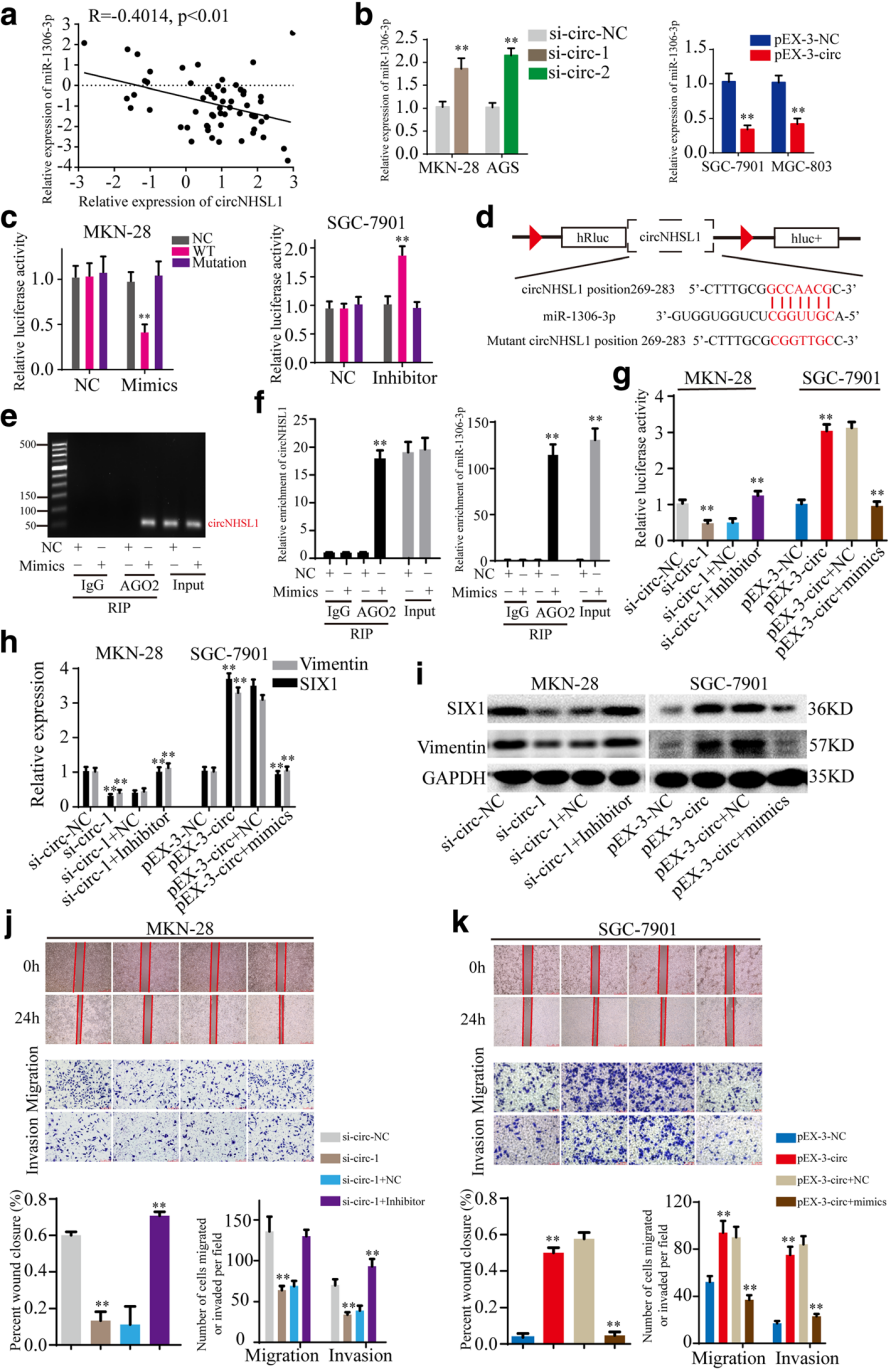
CircNHSL1 promotes gastric cancer progression by serving as a miRNA sponge of miR-1306-3p. **a** Pearson correlation analysis determined the significantly negative correlation between the levels of circNHSL1 and miR-1306-3p in 61 paired gastric cancer tissues (p < 0.01). **b** The effects of circNHSL1 on the expression of SIX1 was detected by qRT-PCR. **c** The effects of mimics, inhibitor and negative control on the luciferase activities were detected in gastric cancer cells after transfecting luciferase reporter plasmids with wild type of WT, Mutant or negative control. **d** Schematic illustration of the sequence of wild type of SIX1 3′-UTR (WT) and mutant sequences on the complementary sites of SIX1 3′-UTR with miR-1306-3p (Mutant). **e** and **f** Anti-AGO2 RIP assay was performed in MKN-28 cells after transfection with mimics or negative control, followed by agarose gel electrophoresis (**e**) and qRT-PCR (**f**) to detect the expression of circNHSL1 and miR-1306-3p. **g** The effects of circNHSL1 and miR-1306-3p on the luciferase activities of wild type of SIX1 mRNA 3′-UTR in gastric cancer cells were detected. **h** and **i** The effects of circNHSL1 and miR-1306-3p on the mRNA and protein expressions of SIX1 and Vimentin were detected by qRT-PCR and western blotting. **j** and **k** The effects of circNHSL1 and miR-1306-3p on the mobility, migration and invasion were confirmed by wound healing and transwell assays. All data are presented as the mean ± SEM of three experiments. **p < 0.01

Previous studies have revealed that circRNAs can serve as miRNA sponges to abrogate the functions of miRNAs. In the following studies, we explored whether circNHSL1 promoted gastric cancer progression by sponging miRNAs. Firstly, we predicted the potential target miRNAs of circNHSL1 with miRanda database (http://www.microrna.org) and the possible upstream miRNAs of SIX1 with TargetScan database (http://www.targetscan.org). The results showed that circNHSL1 and SIX1 share microRNA response elements (MREs) of miR-1306-3p with high scores (Fig. 5d, Additional file 5: Figure S4b). Next, the level of miR-1306-3p was examined in above 61 paired gastric cancer tissues and matched normal tissues, and the results indicated that miR-1306-3p was remarkably down-regulated in 80.33% (49/61) gastric cancer tissues than in normal gastric tissues (Additional file 6: Figure S5a). Pearson correlation analysis showed that the level of circNHSL1 negatively correlated with the level of miR-1306-3p in above gastric cancer tissues (*p* < 0.01) (Fig. 5a). In addition, knockdown or overexpression of circNHSL1 caused up-regulation or down-regulation of miR-1306-3p, respectively, in gastric cancer cells (Fig. 5b). Then, luciferase reporter plasmids with a wild type of circNHSL1 sequence (WT) and mutant circNHSL1 sequence in the binding sites of miR-1306-3p (Mutant) (Fig. 5d) were generated. Luciferase reporter assays illustrated that overexpression of miR-1306-3p suppressed the luciferase activity of WT and knockdown of miR-1306-3p increased the luciferase activity of WT, but did not change the luciferase activity of Mutant (Fig. 5c), demonstrating the direct interaction between circNHSL1 and miR-1306-3p. It has been widely known that miRNAs bind to MREs through RNA-induced silencing complex (RISC), of which Argonaute2 (AGO2) protein is the key component. Thereby, an anti-AGO2 RIP assay was performed in MKN-28 cells to pull down circNHSL1 and miR-1306-3p with an anti-AGO2 antibody (IgG as negative control, none as Input). The results showed that both circNHSL1 and miR-1306-3p were efficiently pulled down by anti-AGO2 antibody compared with IgG, and significantly enriched by overexpression of miR-1306-3p compared with negative control (Fig. 5e and f). Collectively, these data demonstrated that circNHSL1 acts as a sponge of miR-1306-3p in gastric cancer.

### MiR-1306-3p reverses the ability of circNHSL1 to promote gastric cancer progression

To explore whether circNHSL1 exerts its biological function by sponging miR-1306-3p, rescued experiments were performed with up-regulation or down-regulation of miR-1306-3p on the basis of ectopic circNHSL1 expression. Luciferase reporter assays demonstrated that miR-1306-3p inhibitor increased the luciferase activity of the luciferase reporter plasmid with wild type of SIX1 mRNA 3′-UTR, which was suppressed by knockdown of circNHSL1, while miR-1306-3p mimics decreased the luciferase activity induced by overexpression of circNHSL1 (Fig. 5g). Furthermore, miR-1306-3p reversed the ability of circNHSL1 to enhance mRNA and protein expression of SIX1 and Vimentin in gastric cancer cells (Fig. 5h and i). Then, wound healing and transwell migration and invasion assays were conducted. The results showed that miR-1306-3p attenuated the ability of circNHSL1 to promote mobility, migration and invasion of gastric cancer cells (Fig. 5j and k). In summary, circNHSL1 promotes gastric cancer progression through sponging miR-1306-3p.

### MiR-1306-3p is down-regulated in gastric cancer tissues and negatively correlates with clinicopathological features and prognosis

**Fig. 6:**
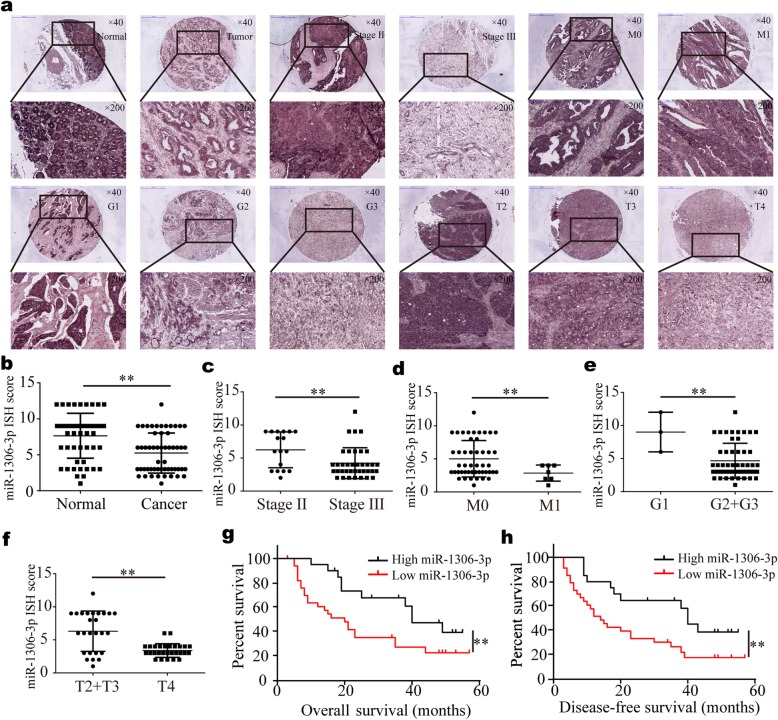
MiR-1306-3p is down-regulated in gastric cancer tissues and correlated with the progression and poor prognosis. The expression of miR-1306-3p was detected by ISH with TMA including 54 paired gastric cancer tissues and paired normal tissues. **a** Represent images of miR-1306-3p expression in normal gastric mucosa and gastric cancer tissues with Stage II, Stage III, M0, M1, G1, G2, G3, pT2, pT3 and pT4. **b** The level of miR-1306-3p in gastric cancer tissues was significantly lower than in normal gastric tissues. **c-f** The level of miR-1306-3p in gastric cancer tissues with Stage II (**c**), M0 (**d**), well differentiation (**e**) and pT2 and pT3 (**f**) was significantly higher than these with Stage III, M1, moderate and poor differentiation and pT4. **g**-**h** Kaplan-Meier survival analysis (log-rank test) showed that gastric cancer patients with low miR-1306-3p expression have a lower OS and DFS than these with high miR-1306-3p expression (*p* < 0.01). All data are presented as the mean ± SEM. **p < 0.01

Few studies about miR-1306-3p have been reported, and the expression level, functions and roles of miR-1306-3p in cancers have not been explored. Therefore, miR-1306-3p has yet to be understood. In this study, we used another group of gastric cancer tissues (a TMA including 54 paired gastric cancer tissues and matched normal tissues) to detect the level of miR-1306-3p with ISH. The results indicated that consistent with qRT-PCR results in above 61 paired fresh frozen gastric cancer tissues, the level of miR-1306-3p was significantly higher in normal gastric tissues than in gastric cancer tissues (Fig. 6a and b). Further analysis showed that miR-1306-3p was markedly higher in gastric cancer tissues of UICC stage II, distant metastasis M0 stage, well differentiation (G1 stage) and pathological T2 and T3 stages than in these of UICC stage III, distant metastasis M1 stage, moderate and poor differentiation (G2 and G3 stages) and pathological T4 stage, respectively (Fig. 6c-f). MiR-1306-3p expression in gastric cancer tissues negatively correlated with differentiation, pathological T stage, distant metastasis and UICC stage (Table 2). The Kaplan-Meier analysis showed that gastric cancer patients with low miR-1306-3p expression had a shorter OS and DFS (Fig. 6g and h), indicating low miR-1306-3p expression predicted a poor prognosis. In general, miR-1306-3p acts as a tumor suppressor and high miR-1306-3p predicts a promising prognosis in gastric cancer.

**Table 2:** Correlation between miR-1306-3p expression and clinicopathological parameters in gastric cancer (*n* = 54)

Parameters	Category	No.	miR-1306-3p expression		*χ* ^*2*^	P
			Low	High		
Age					0.845	0.358
	< 65	26	18	8		
	≥65	28	16	12		
Gender					0.327	0.568
	Male	38	23	15		
	Female	16	11	5		
T stage					19.703	< 0.001
	T2 + T3	26	8	18		
	T4	28	26	2		
N stage					2.000	0.157
	N0+ N1	23	12	11		
	N2+ N3	31	22	9		
M stage						0.030
	M0	47	27	20		
	M1	7	7	0		
UICC stage					8.145	0.004
	II	17	6	11		
	III	37	28	9		
Nerve invasion					4.469	0.035
	Yes	29	22	7		
	No	25	12	13		
Vessel invasion					0.021	0.884
	Yes	29	16	9		
	No	25	18	11		
Differentiation						0.046
	Well	3	0	3		
	Mederate+ Poor	51	34	17		
Toumor size					0.454	0.561
	≤3 cm	23	15	7		
	> 3 cm	31	18	13		
Tumor		54	34	20	18.328	< 0.001
Normal		54	12	42		

### SIX1 is a direct target of miR-1306-3p

**Fig. 7:**
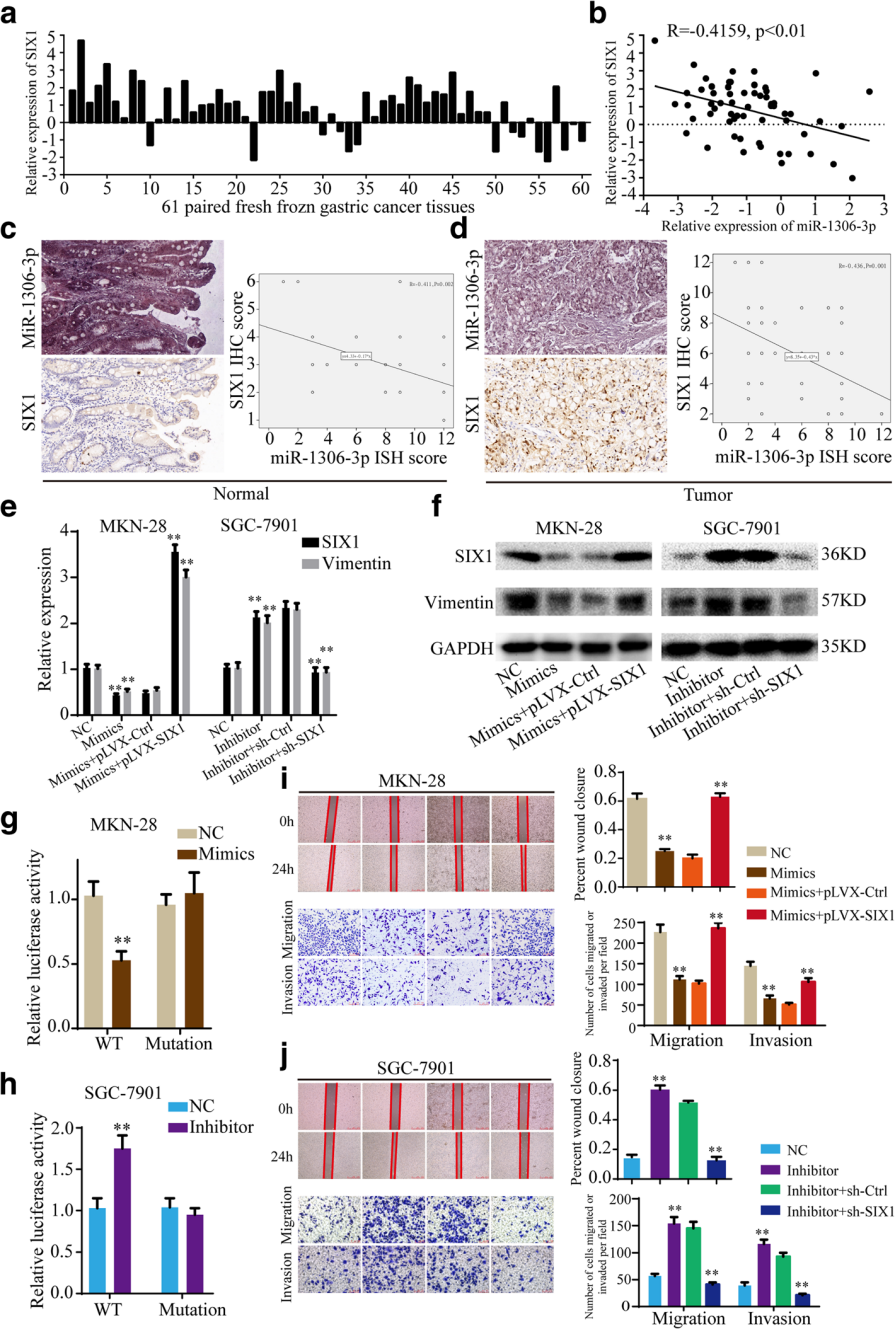
MiR-1306-3p suppresses gastric cancer progression through directly targeting SIX1. **a** SIX1 expression is up-regulated in 77.05% (47/61) gastric cancer tissues. **b** Pearson correlation analysis determined the significantly negative correlation between the levels of miR-1306-3p and SIX1 in 61 paired gastric cancer tissues (*p* < 0.01). **c** and **d** The ISH scores of miR-1306-3p negatively correlated with the IHC scores of SIX1 in both normal gastric mucosa tissues (**c**) and gastric cancer tissues (**d**) of TMA. **e** and **f** The effects of miR-1306-3p and SIX1 on the mRNA and protein expressions of SIX1 and Vimentin were detected by qRT-PCR (**e**) and western blotting (**f**). **g** and **h** The effects of mimics (**g**) and inhibitor (**h**) of miR-1306-3p on the luciferase activities of wild type of SIX1 mRNA 3′-UTR (WT) and mutant SIX1 mRNA 3′-UTR (Mutant) were detected. **i** and **j** The effects of miR-1306-3p and SIX1 on the mobility, migration and invasion were detected by wound healing and transwell assays. All data are presented as the mean ± SEM of three experiments. **p < 0.01

According to the TargetScan database, SIX1 mRNA contains the MRE of miR-1306-3p, implying that miR-1306-3p may direct target SIX1. We analyzed the expression of miR-1306-3p and SIX1 in above 61 paired gastric cancer tissues, and found SIX1 was up-regulated in 77.05% (47/61) gastric cancer tissues (Fig. 7a) and the level of miR-1306-3p negatively correlated with the level of SIX1 (Fig. 7b). Then, the level of SIX1 was determined in above mentioned TMA of gastric cancer using immunohistochemistry. Pearson correlation analysis indicated that miR-1306-3p ISH score negatively correlated with SIX1 IHC score in both gastric cancer tissues and normal gastric tissues (Fig. 7c and d). In addition, overexpression of miR-1306-3p significantly decreased the expression of SIX1 mRNA and protein, while down-regulation of miR-1306-3p remarkably increased it, in MKN-28 and SGC-7901 cells (Fig. 7e and f, Additional file 7: Figure S6a and b). Furthermore, luciferase reporter plasmids with the wild type of SIX1 mRNA 3′-UTR (WT) and mutant SIX1 mRNA 3′-UTR in the binding sites of miR-1306-3p (Mutant) were constructed (Additional file 5: Figure S4c). Luciferase reporter assays showed miR-1306-3p mimics significantly decreased the luciferase activity of WT, while miR-1306-3p inhibitor remarkably increased it, but not that of Mutant, in MKN-28 and SGC-7901 cells (Fig. 7g and h, Additional file 7: Figure S6c and d). These data suggested that miR-1306-3p suppresses SIX1 expression by directly binding to 3′-UTR of SIX1 mRNA.

### MiR-1306-3p suppresses gastric cancer progression through inhibiting SIX1 expression

Given the roles of circNHSL1 and SIX1 on promoting gastric cancer progression, we detected the role of miR-1306-3p on the migration and invasion of gastric cancer cells. The results showed that miR-1306-3p mimics suppressed the mobility, migration and invasion of MKN-28 and SGC-7901 cells (Fig. 7i, Additional file 7: Figure S6f), while miR-1306-3p inhibitor enhanced the mobility, migration and invasion of gastric cancer cells (Fig. 7j, Additional file 7: Figure S6e).

To further explore the effects of SIX1 on the roles of miR-1306-3p in gastric cancer progression, rescued experiments were conducted. As shown in Fig. 7e and f, Additional file 7: Figure S6a and b, SIX1 reversed the ability of miR-1306-3p to suppress the mRNA and protein level of SIX1 and Vimentin. Functionally, SIX1 reversed the ability of miR-1306-3p to inhibit the mobility, migration and invasion of gastric cancer cells (Fig. 7i and j, Additional file 7: Figure S6e and f). To summary, miR-1306-3p suppresses gastric cancer progression through inhibiting SIX1 expression.

### CircNHSL1 enhances the growth and metastasis of xenograft tumors of gastric cancer cells in vivo

**Fig. 8:**
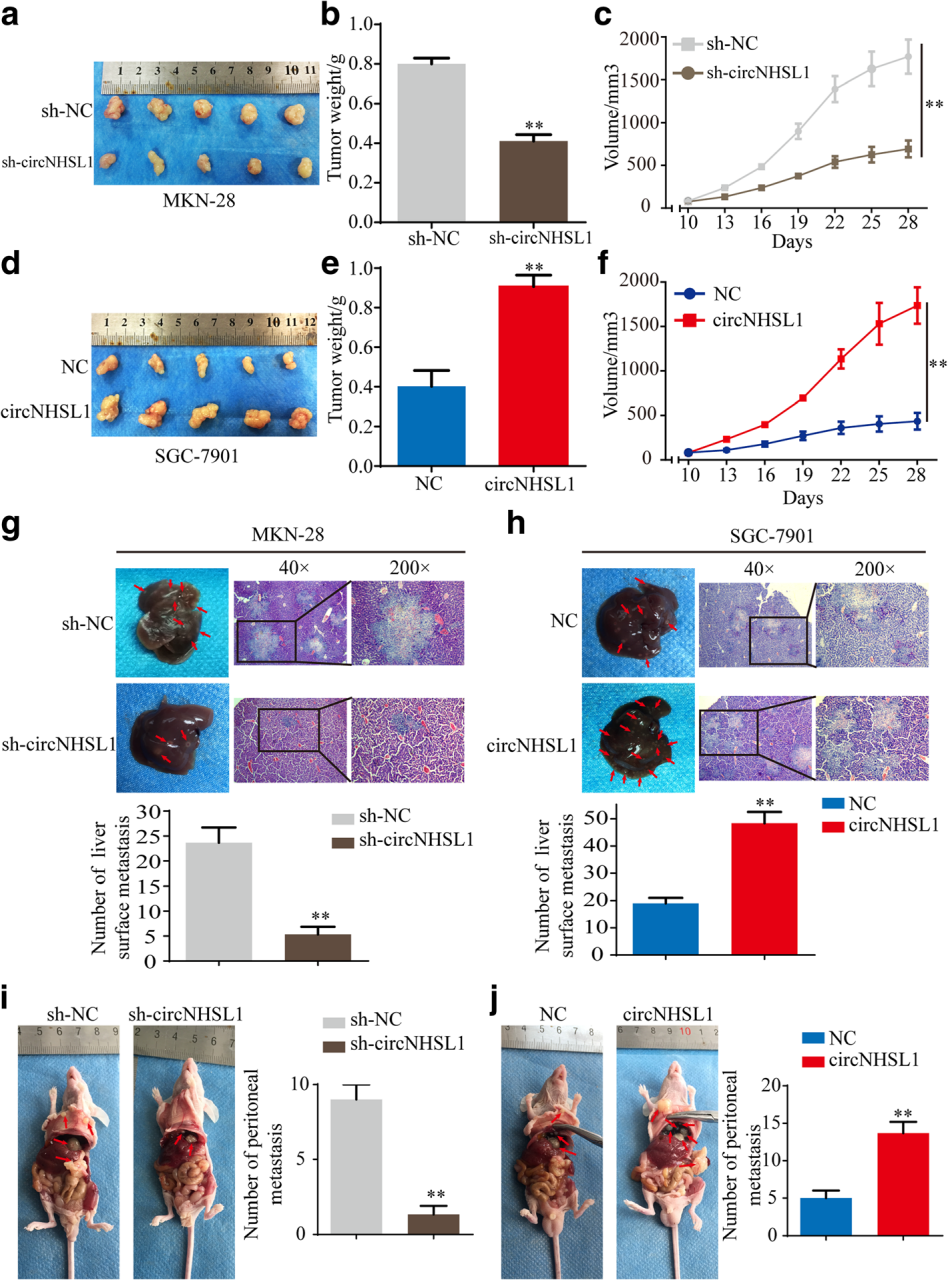
CircNHSL1 promotes growth and metastasis of gastric cancer cells in vivo. **a** Images of subcutaneous xenograft tumors of MKN-28 cells. **b** Tumor weight of MKN-28 cells was shown. **c** Tumor volumes of MKN-28 cells measured every 3 days for 6 times were analyzed. **d** Image of subcutaneous xenograft tumors of SGC-7901 cells. **e** Tumor weight of SGC-7901 cells was shown. **f** Tumor volumes of SGC-7901 cells measured every 3 days for 6 times were analyzed. **g** Representative images of liver metastasis assay and HE staining of the liver of MKN-28 cells. Knockdown of circNHSL1 decreased the number of liver surface metastasis compared with negative control. **h** Representative images of liver metastasis assay and HE staining of the liver of SGC-7901 cells. Overexpression of circNHSL1 increased the number of liver surface metastasis compared with negative control. **i-j** Representative images of peritoneal metastasis assays. Knockdown of circNHSL1 decreased the number of peritoneal metastasis nodules compared with negative control (**i**), whereas overexpression of circNHSL1 increased the number of peritoneal metastasis nodules (**j**). **p < 0.01

To investigate the functions of circNHSL1 in vivo, the stable MKN-28 cells with sh-circNHSL1 or sh-NC and SGC-7901 cells with circNHSL1 or NC were constructed, according to circNHSL1 expression in 7 gastric cancer cells. The xenograft mouse model was established by subcutaneously injecting of gastric cancer cells. After 28 days, all mice were sacrificed and tumor samples were harvested (Fig. 8a and d). The weight (Fig. 8b) and volume (Fig. 8c) of tumors with knockdown of circNHSL1 were markedly lower than those with control in MKN-28 cells, while the weight (Fig. 8e) and volume (Fig. 8f) of tumors with overexpression of circNHSL1 were significantly higher than the control tumors of SGC-7901 cells.

The role of circNHSL1 on metastasis was confirmed by in vivo liver metastasis and peritoneal metastasis assays. We observed that the number of liver surface metastatic nodules in mice bearing MKN-28 cells with sh-circNHSL1 was lower than in mice bearing MKN-28 cells with sh-NC (Fig. 8g), whereas the number of liver surface metastatic nodules in mice bearing SGC-7901 cells with circNHSL1 was much than in mice bearing SGC-7901 cells with NC (Fig. 8h). Necropsy revealed that the number of peritoneal metastatic nodules was reduced in mice bearing MKN-28 cells with sh-circNHSL1 compared with sh-NC (Fig. 8i), while SGC-7901 cells with circNHSL1 colonized the visceral organs and formed multiple metastatic nodules (Fig. 8j), revealing that circNHSL1 enhanced tumor colonization and metastasis. Overall, these data demonstrate that circNHSL1 promotes cell growth and metastasis of gastric cancer in vivo.

## Discussion

CircRNAs are a new type of highly stable and abundant endogenous noncoding RNAs. With the development of high-throughput sequencing and bioinformatics analysis, an increasing number of circRNAs have been identified and confirmed to regulate the development and progression of various human cancers in recent years [31–33]. However, few circRNAs have been well functionally and mechanistically characterized in gastric cancer, and the biological functions of most circRNAs have yet to be explored. In this study, we identified a novel metastasis-related circRNA circNHSL1, which is dramatically up-regulated in gastric cancer tissues and cells.

Invasion and metastasis are the biggest obstacles to successful surgical resection of tumors, as well as the leading cause of high mortality of gastric cancer patients [34]. Hence, it is urgent to discover metastasis-related genes and illustrate the molecular mechanisms of invasion and metastasis of gastric cancer. In the present study, we applied RNA-seq analysis to access the expression profile of metastasis-related circRNA between 3 gastric cancer tissues without metastasis and 2 gastric cancer tissues with metastasis. The most highly up-regulated circRNA in 2 gastric cancer tissues with metastasis, circNHSL1, was further confirmed to be obviously high in 61 paired gastric cancer tissues and positively correlate with pathological T stages, lymphatic metastasis, distant metastasis and poor prognosis. Functionally, circNHSL1 promotes invasion and metastasis of gastric cancer cells in vitro and in vivo, implying that circNHSL1 is a tumor promoter in gastric cancer. Due to the stable loop structure, great resistance to exoribonuclease and high abundance in the cytoplasm, circNHSL1 may be an efficient diagnostic and therapeutic target and a promising biomarker for prognosis in gastric cancer.

CircRNAs have been reported to have multiple and diverse molecular mechanisms in the development and progression of various cancers, among which ceRNA is the most important and frequently reported. CeRNA hypothesis suggests that circRNAs serve as ceRNAs to positively regulate the expression of miRNA target genes [35, 36]. For example, circAKT3 promotes PIK3R1 expression via miR-198 suppression in gastric cancer [37]. CircPSMC3 acts as a miR-296-5p sponge to promote the expression of Phosphatase and Tensin Homolog (PTEN) [38]. In the present study, RNA-seq between above 3 gastric cancer tissues without metastasis and 2 gastric cancer tissues with metastasis used in RNA-seq for circRNAs was performed to analyze metastasis-related genes. The top 10 genes of high expression in 2 gastric cancer tissues with metastasis were selected. A series of molecular experiments demonstrated that circNHSL1 promotes SIX1 expression in gastric cancer. Subsequent rescued experiments confirmed that overexpression of SIX1 reverse the ability of decreased circNHSL1 to suppress the mobility, migration and invasion, while down-regulation of SIX1 attenuated the ability of enhanced circNHSL1 to promote the mobility, migration and invasion. In summary, circNHSL1 promotes malignance of gastric cancer cells through enhancing SIX1 expression.

The ceRNA hypothesis constructs a complicated regulatory network and mechanism. CircRNAs contain one or more MREs that act as miRNA sponges to negatively modulate miRNA activity, attenuating the inhibitory effect on their target genes. Indeed, an increasing amount of evidence demonstrated the posttranscriptional function of circRNAs. The most well-known exonic cricRNA CDR1as/ciRS-7 has been demonstrated to contain 63 or 70 binding sites for miR-7 and considered as one of the most powerful miRNA sponge [29, 30]. CircNRIP1 activates AKT1/mTOR pathway by sponging miR-149-5p in gastric cancer [39]. In addition, circRNA-MYLK promotes cell proliferation, migration and invasion by directly binding to miR-29a and subsequently relieves suppression for target VEGFA, which activates VEGFA/VEGFR2 signaling pathway in bladder cancer [40]. Here, bioinformatics analysis showed that circNHSL1 and SIX1 share MRE of miR-1306-3p, implying the formation of circNHSL1/miR-1306-3p/SIX1 axis. Luciferase reporter and RIP assays confirmed the direct interaction between circNHSL1 and miR-1306-3p. Likewise, loss and gain and luciferase reporter assays demonstrated that miR-1306-3p directly suppresses SIX1 expression through binding to 3′-UTR of SIX1 mRNA. Due to the unknown levels and functions of miR-1306-3p in tumors, we detected the expression levels and functions of miR-1306-3p in gastric cancer. We first demonstrated that miR-1306-3p was down-regulated in gastric cancer tissues, negatively correlated with clinicalpathological features and poor prognosis and suppressed the progression of gastric cancer, implying that miR-1306-3p may act as a tumor suppressor. Furthermore, miR-1306-3p reverses the ability of circNHSL1 to promote SIX1 expression and the mobility, migration and invasion, while SIX1 reverses the ability of miR-1306-3p to suppress the mobility, migration and invasion. Combining previous results, we demonstrate that circNHSL1 promotes gastric cancer progression through serving as a miR-1306-3p sponge and relieving its suppression on target gene SIX1 expression.

SIX1 is a transcription factor of the homeobox gene family. Previous studies have reported that SIX1 plays important roles in the development and progression of multiple cancers [41, 42]. SIX1 is up-regulated in pancreatic cancer tissues and promotes cell migration and invasion in vitro and growth in vivo [43]. SIX1 is also up-regulated in colorectal cancer, correlates with poor overall survival and promotes cancer cell growth and metastasis in vitro and in vivo [44]. Additionally, as a transcription factor, SIX1 enhances VEGF-C and ZEB1 expression to promote EMT, invasion and metastasis of cancer cells [26]. Nevertheless, the detail functions and mechanisms of SIX1 in gastric cancer remain unknown. We detected that SIX1 is up-regulated in gastric cancer tissues, and promotes the mobility, migration and invasion of gastric cancer cells. Bioinformatics analysis indicated that the promoter domain of Vimentin, a key mesenchymal marker that promotes cell EMT, invasion and metastasis [27], contains two binding sites of SIX1. Further molecular experiments demonstrated that SIX1 transcriptionally promotes Vimentin expression through directly binding to the promoter domain of Vimentin. Taken together, circNHSL1 promotes gastric cancer progression by miR-1306-3p/SIX1/Vimentin axis.

It is widely accepted that circRNAs exist in different species, possessing the features of broadly evolutionary conservation, high abundance, tissue specificity and space-time specificity. The discovery of the novel circRNA circNHSL1 would be enlightening. It has been reported that some circRNAs exist stably in plasma and exosomes [9, 45, 46], which makes it possible to be biomarkers and targets for diagnosis, prognosis and therapy in certain diseases. Therefore, whether circNHSL1 can be detected in plasma and exosomes needs further investigation. In addition, one circRNA may contain multiple different miRNA binding sites, and one miRNA can also bind to several circRNAs. Hence, circRNAs and miRNAs may crosstalk with each other in biological processes. Moreover, lots of unknown circRNAs and their functions in the development and progression of cancers remain to be discovered. Accordingly, more endeavors and further studies are needed to reveal the functions and mechanisms of circRNAs in cancers.

## Conclusion

In summary, we identified that a novel metastasis-related circRNA circNHSL1 is up-regulated in gastric cancer tissues and positively correlates with clinicopathological features and poor prognosis. Furthermore, we demonstrated that circNHSL1 promotes invasion and metastasis of gastric cancer cells in vitro and in vivo by targeting the miR-1306-3p/SIX1/Vimentin axis. Our findings firstly identify the role of circNHSL1, which may offer an effective biomarker for diagnosis and prognosis and a promising target for therapy in gastric cancer.

## Additional files

Additional file 1: **Table S1.** The sequences of primers for qRT-PCR. (DOCX 17 kb)

Additional file 2: **Figure S1.** The relative expression of circNHSL1 in 93 paired fresh frozen normal gastric tissues and gastric cancer tissues. CircNHSL1 expression was significantly higher in most (80.65%, 75/93) gastric cancer tissues than in normal gastric tissues. (TIF 180 kb)

Additional file 3: **Figure S2.** CircNHSL1 promotes migration and invasion of gastric cancer cells in vitro. a and b Relative expression of circNHSL1 and NHSL1 mRNA was detected by qRT-PCR in gastric cancer cells after transfection of si-circNHSL1, pEX-3-circNHSL1 or negative control. c and d The cell mobility, migration and invasion were evaluated by wound healing and transwell migration and invasion assays after overexpression of circNHSL1 in MKN-28 cells. e and f The cell mobility, migration and invasion were evaluated by wound healing and transwell assays after knockdown of circNHSL1 in SGC-7901 cells. All data are presented as the mean ± SEM of three experiments. **p < 0.01. (TIF 5301 kb)

Additional file 4: **Figure S3.** The effects of circNHSL1 on the expression of the top 10 gene candidates. a The effects of knockdown of circNHSL1 on the expression of the top 10 gene candidates in AGS cells. b The effects of overexpression of circNHSL1 on the expression of the top 10 gene candidates in MGC-803 cells. c and d The effects of knockdown and overexpression of circNHSL1 on the expression of SIX1 mRNA (c) and protein (d) in AGS and MGC-803 cells. (TIF 381 kb)

Additional file 5: **Figure S4.** Schematic illustration of the complementary sites of SIX1 mRNA 3′-UTR with miR-1306-3p. a The potential binding DNA sequence logo of SIX1 protein. b The prediction of binding sites of SIX1 mRNA 3′-UTR with miR-1306-3p based on TargetScan database. c The luciferase reporter plasmids containing the wild type of SIX1 mRNA 3′-UTR (WT) and mutant sequence in the binding sites of SIX1 mRNA 3′-UTR with miR-1306-3p (Mutant) were constructed. (TIF 252 kb)

Additional file 6: **Figure S5.** The expression of miR-1306-3p. a The level of miR-1306-3p was down-regulated in 80.33% (49/61) gastric cancer tissues. b The efficiencies of transfection with mimics and inhibitor of miR-1306-3p were determined in MKN-28 and SGC-7901 cells. (TIF 272 kb)

Additional file 7: **Figure S6.** MiR-1306-3p suppresses gastric cancer progression through directly targeting SIX1. a and b The effects of miR-1306-3p and SIX1 on the mRNA and protein expressions of SIX1 and Vimentin were detected by qRT-PCR (**a**) and western blotting (b). c and d The effects of inhibitor (c) and mimics (d) of miR-1306-3p on the luciferase activities of wild type of SIX1 mRNA 3′-UTR (WT) and mutant SIX1 mRNA 3′-UTR (Mutant) were detected in MKN-28 and SGC-7901 cells. e and f The effects of miR-1306-3p and SIX1 on the mobility, migration and invasion were detected by wound healing and transwell assays in MKN-28 and SGC-7901 cells. All data are presented as the mean ± SEM of three experiments. **p < 0.01. (TIF 5078 kb)

## Data Availability

The datasets used for the current study are available from the corresponding author on reasonable request.

## Abbreviations

3’UTR: 3′ -untranslated region
ceRNA: Competing endogenous RNA
CircRNA: Circular RNA
DFS: Disease-free survival
EMT: Epithelial–mesenchymal transition
H&E: Hematoxylin and eosin
IHC: Immunohistochemical analysis
ISH: In situ hybridization
miRNA: MicroRNA
MREs: microRNA response elements
NHSL1: the NHS like-1
OS: Overall survival
PTEN: Phosphatase and Tensin Homolog
PVDF: Polyvinylidene fluoride
qRT-PCR: Real-time quantitative polymerase chain reaction
RIP: RNA immunoprecipitation
RPL26: Ribosomal protein L26
SIX1: Sine oculis homeobox homolog 1
TMA: Tissue microarray
UICC: the Union for International Cancer Control
