# Correction to: Circular RNA circNHSL1 promotes gastric cancer progression through the miR-1306-3p/SIX1/Vimentin axis

**DOI:** 10.1186/s12943-020-01164-y

**Published:** 2020-03-27

**Authors:** Zhonglin Zhu, Zeyin Rong, Zai Luo, Zhilong Yu, Jing Zhang, Zhengjun Qiu, Chen Huang

**Affiliations:** Department of General Surgery, Shanghai General Hospital, Shanghai Jiaotong University School of Medicine, 650 Xinsongjiang Road, Songjiang District, Shanghai, 201600 China

**Correction to: Mol Cancer (2019) 18:126**

**https://doi.org/10.1186/s12943-019-1054-7**

After publication of the article [1], the authors reported errors of inter-duplication in Fig. 3. As shown in Fig. 3, the “Transwell assay” images of “pEX-3-circ+LV-shCtrl” and “pEX-3-circ+LV-shSIX1” group in Fig. 3j were shown identical to “pEX-3-circ+NC” and “pEX-3-circ+mimics” group in Fig. 5k. The authors have confirmed that the “Transwell assay” images of “pEX-3-circ+LV-shCtrl” and “pEX-3-circ+LV-shSIX1” group were mistakenly presented in the original Fig. 3j. This mistake was caused by unintentionally covering the correct image, which is reflected by the histograms and its original data. The “Migration assay” image of “si-circ-1” in Fig. 3i contained an inter-duplication in error with the image of “si-circ-1 + pLVX-SIX1” in Fig. 5j, which was caused by the misplacement of the list of the “Migration assay” images in Fig. 3i. In addition, the “Migration assay” images in Fig. 4 h and Fig. S2d were inadvertently misplaced by the same reason. The histograms are correct and reflect the accuracies of the results. The errors do not change the conclusion or discussion of the article. The authors apologize for any inconvenience caused by unintentional misplace.

The correct figures are updated below.

**Fig.3i** and **j**

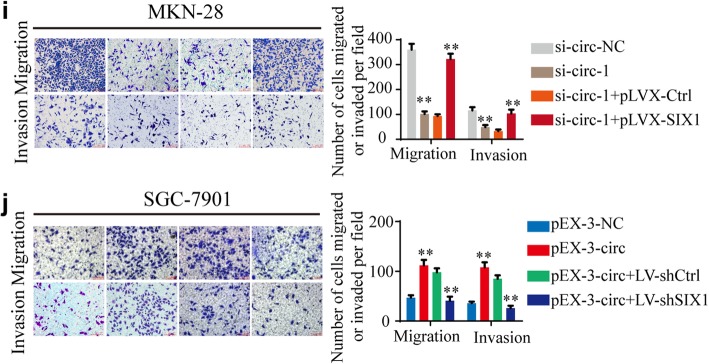



**Fig.4h**

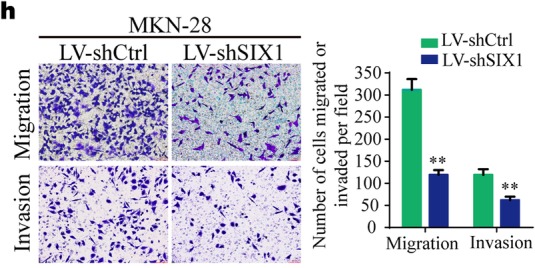



Additional file 3: Figure S2d


