# Supplementary material for: Circular RNA circNHSL1 promotes gastric cancer progression through the miR-1306-3p/SIX1/vimentin axis
Source: Mol Cancer. 2019 Aug 22;18:126. doi: 10.1186/s12943-019-1054-7 (PMC6704702; doi:10.1186/s12943-019-1054-7)
Supplement: Supplementary file 1 — Table S1. The sequences of primers for qRT-PCR. (DOCX 17 kb) [file 12943_2019_1054_MOESM1_ESM.docx]

Table S1. The sequences of primers for qRT-PCR.

| CircNHSL1 | Sense  Antisense | AGCTAGGACTACAGTCTTTCCAGTG GAATGGATCTGTCCGTTTACTATTG |
| --- | --- | --- |
| SIX1 | Sense  Antisense | CGTGCGTGTGCGAGGTTCTG  TGGTGGCTCTCCAGGATCTTGTAG |
| Vimentin | Sense  Antisense | TTGCCGTTGAAGCTGCTAACTACC  AATCCTGCTCTCCTCGCCTTCC |
| NHSL1 | Sense  Antisense | GATTCTGCAGGGATCGTATTCC  TAGAGGAAAGTGCCATTCATGT |
| NR4A2 | Sense  Antisense | CTATGACCAGCCTGGACTATTC  GCCAGTCAGGAGATCATAGAAT |
| ROR2 | Sense  Antisense | CAATGTGCTAGTGTACGACAAG  CTTGTAGTAATCGGCGGCATA |
| MAN2C1 | Sense  Antisense | GTCGTATTGGAGACCGTCAAG  CTTGGAAGGGAGAAAAGGTGAG |
| PHLDB3 | Sense  Antisense | CTGAGGATCGAGATGGAGGTGGAG  AGCCGCCTCTGTTCCTGTTCC |
| ELMO2 | Sense  Antisense | GGGACAATCTTACAACTGGCTA  CATGTTGATGAACTCAGTAGCG |
| AMOT | Sense  Antisense | TGCTGCTGCTGCTGCTGTTC  GCTGAAGTTGGTGCCTGAGTCTG |
| MAP4K4 | Sense  Antisense | ACATGTTAAAACGGGTCAGTTG  GGAGGGCTCTTTTTGATGAAAG |
| CCDC88A | Sense  Antisense | CAAACAGTGTCTTCCTTAAGGC  CTTCAGCTCGTTCTCCTTTTTC |
| MEF2D | Sense  Antisense | GGCTGTCGCTAGGCAATGTCAC  CTGTGGCTGTGGCTGCTGTG |
| GAPDH | Sense  Antisense | GGGAAGGTGAAGGTCGGAGT  GGGGTCATTGATGGCAACA |
| miR-1306-3p | stem-loop  Sense  Antisense | GTCGTATCCAGTGCAGGGTCCGAGGTATTCGCACTGGATACGACTGCAAC  CGCGGTGGTGGTCTCG  AGTGCAGGGTCCGAGGTATT |
| U6 | stem-loop  Sense  Antisense | CTCAACTGGTGTCGTGGAGTCGGCAATTCAGTTGAGAAAAATAT  CAAGGATGACACGCAAA  TCAACTGGTGTCGTGG |
