# Supplementary figures and images for: Circular RNA circNHSL1 promotes gastric cancer progression through the miR-1306-3p/SIX1/vimentin axis
Source: Mol Cancer. 2019 Aug 22;18:126. doi: 10.1186/s12943-019-1054-7 (PMC6704702; doi:10.1186/s12943-019-1054-7)

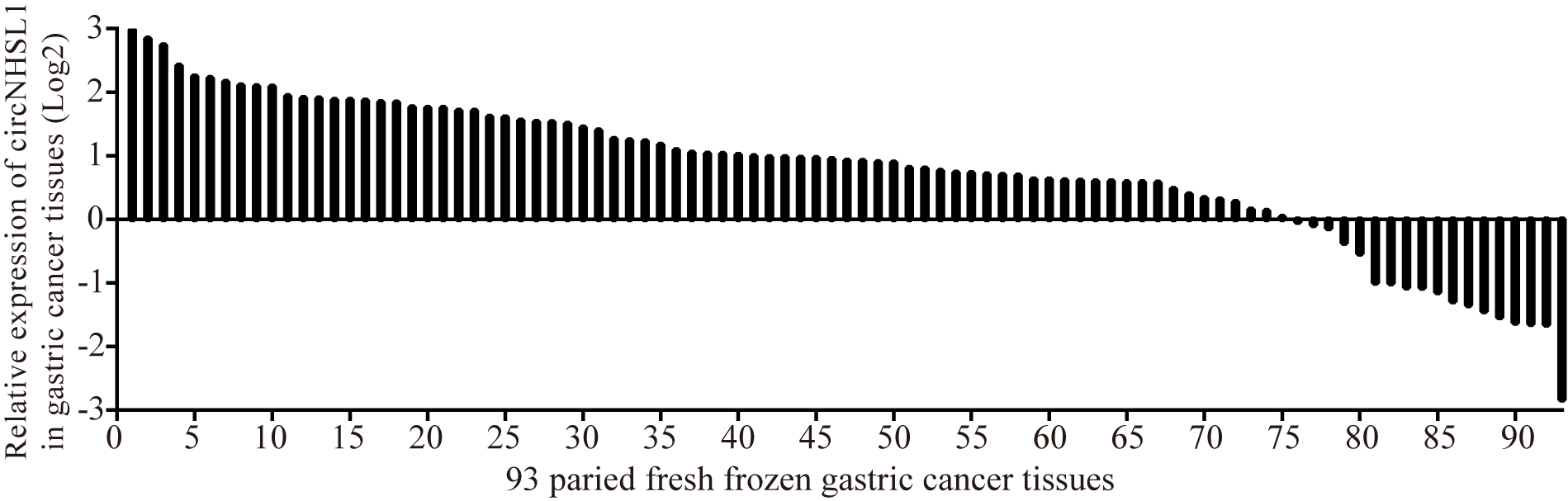

Supplement: Supplementary file 2 — Figure S1. The relative expression of circNHSL1 in 93 paired fresh frozen normal gastric tissues and gastric cancer tissues. CircNHSL1 expression was significantly higher in most (80.65%, 75/93) gastric cancer tissues than in normal gastric tissues. (TIF 180 kb) [file 12943_2019_1054_MOESM2_ESM.tif]

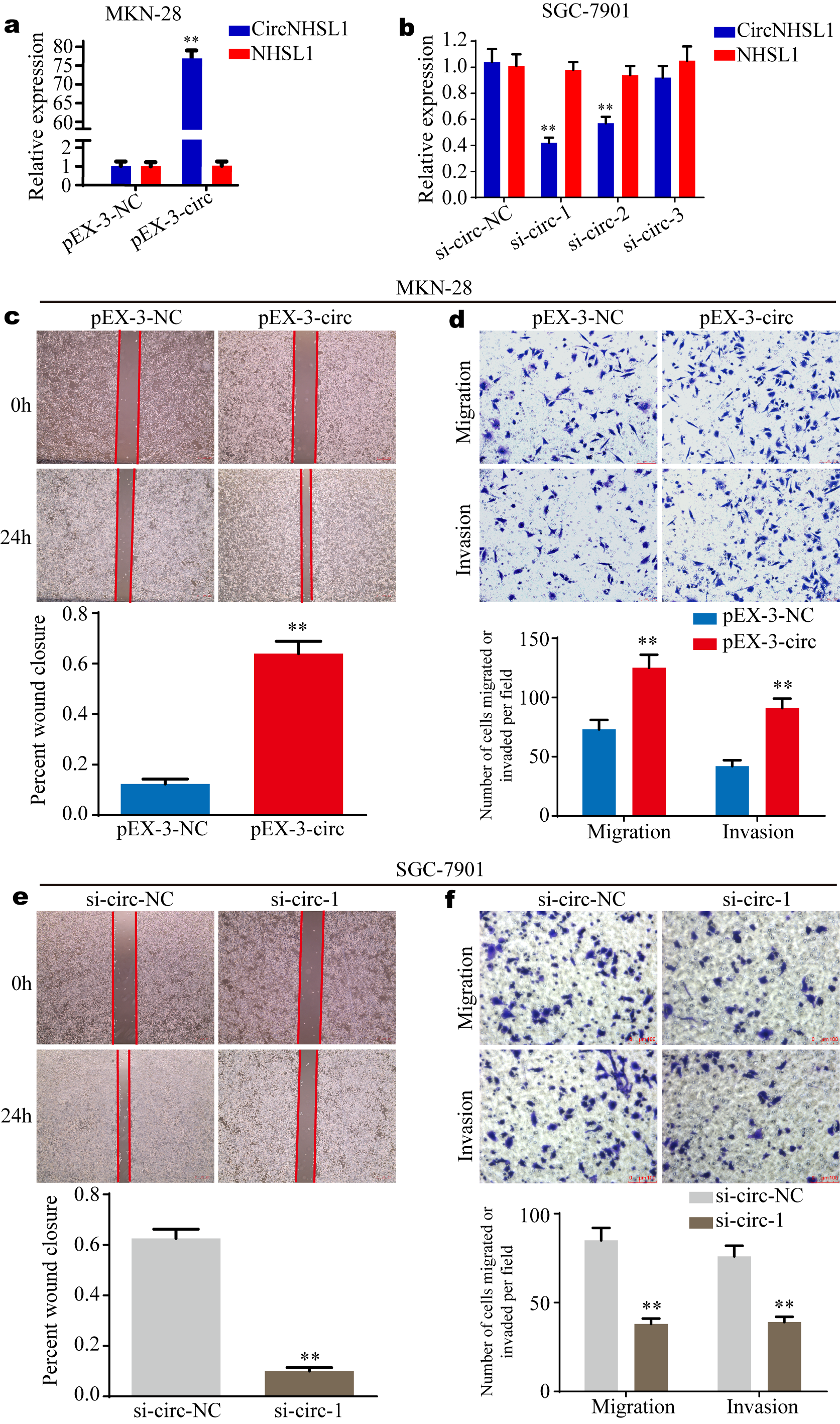

Supplement: Supplementary file 3 — Figure S2. CircNHSL1 promotes migration and invasion of gastric cancer cells in vitro. a and b Relative expression of circNHSL1 and NHSL1 mRNA was detected by qRT-PCR in gastric cancer cells after transfection of si-circNHSL1, pEX-3-circNHSL1 or negative control. c and d The cell mobility, migration and invasion were evaluated by wound healing and transwell migration and invasion assays after overexpression of circNHSL1 in MKN-28 cells. e and f The cell mobility, migration and invasion were evaluated by wound healing and transwell assays after knockdown of circNHSL1 in SGC-7901 cells. All data are presented as the mean ± SEM of three experiments. **p < 0.01. (TIF 5301 kb) [file 12943_2019_1054_MOESM3_ESM.tif]

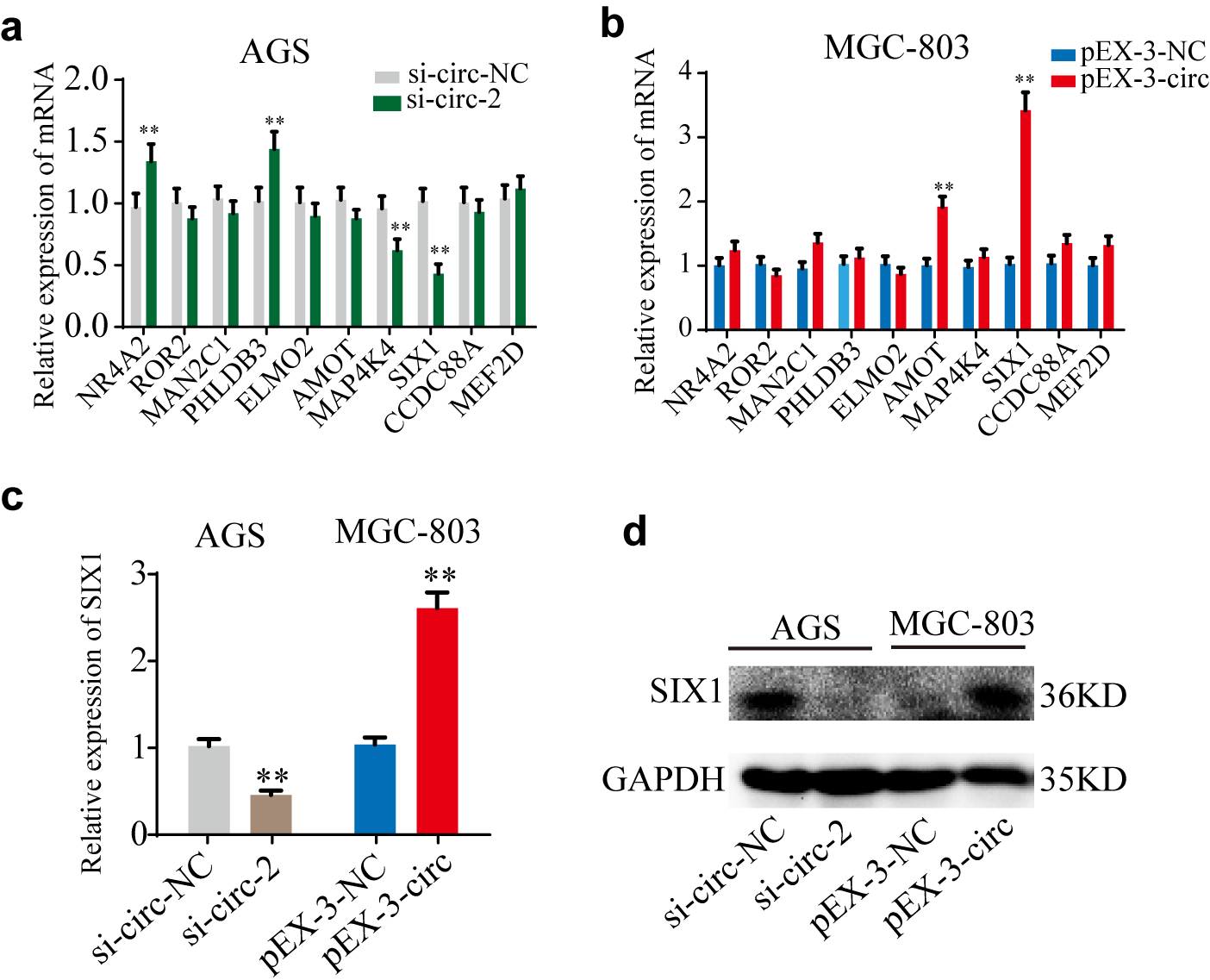

Supplement: Supplementary file 4 — Figure S3. The effects of circNHSL1 on the expression of the top 10 gene candidates. a The effects of knockdown of circNHSL1 on the expression of the top 10 gene candidates in AGS cells. b The effects of overexpression of circNHSL1 on the expression of the top 10 gene candidates in MGC-803 cells. c and d The effects of knockdown and overexpression of circNHSL1 on the expression of SIX1 mRNA (c) and protein (d) in AGS and MGC-803 cells. (TIF 381 kb) [file 12943_2019_1054_MOESM4_ESM.tif]

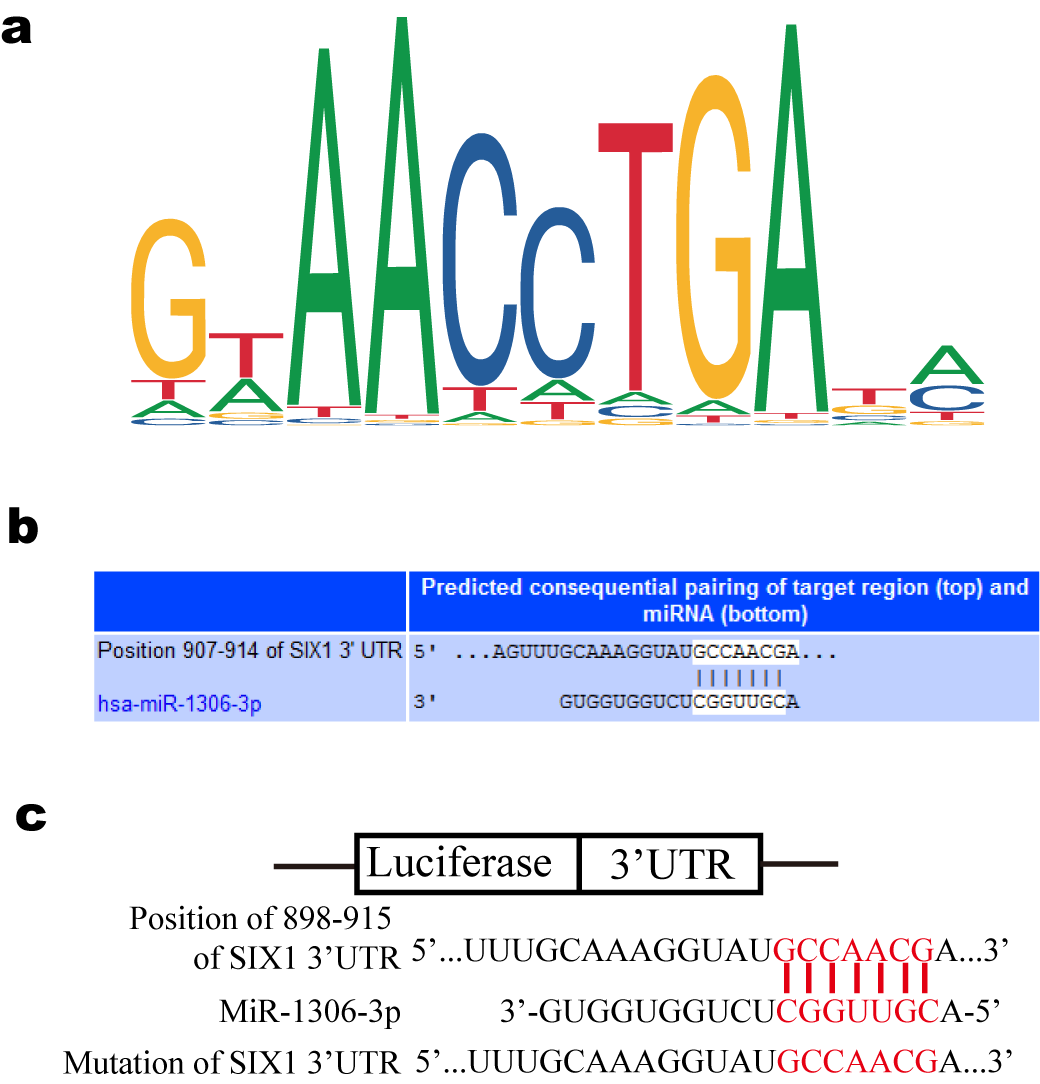

Supplement: Supplementary file 5 — Figure S4. Schematic illustration of the complementary sites of SIX1 mRNA 3′-UTR with miR-1306-3p. a The potential binding DNA sequence logo of SIX1 protein. b The prediction of binding sites of SIX1 mRNA 3′-UTR with miR-1306-3p based on TargetScan database. c The luciferase reporter plasmids containing the wild type of SIX1 mRNA 3′-UTR (WT) and mutant sequence in the binding sites of SIX1 mRNA 3′-UTR with miR-1306-3p (Mutant) were constructed. (TIF 252 kb) [file 12943_2019_1054_MOESM5_ESM.tif]

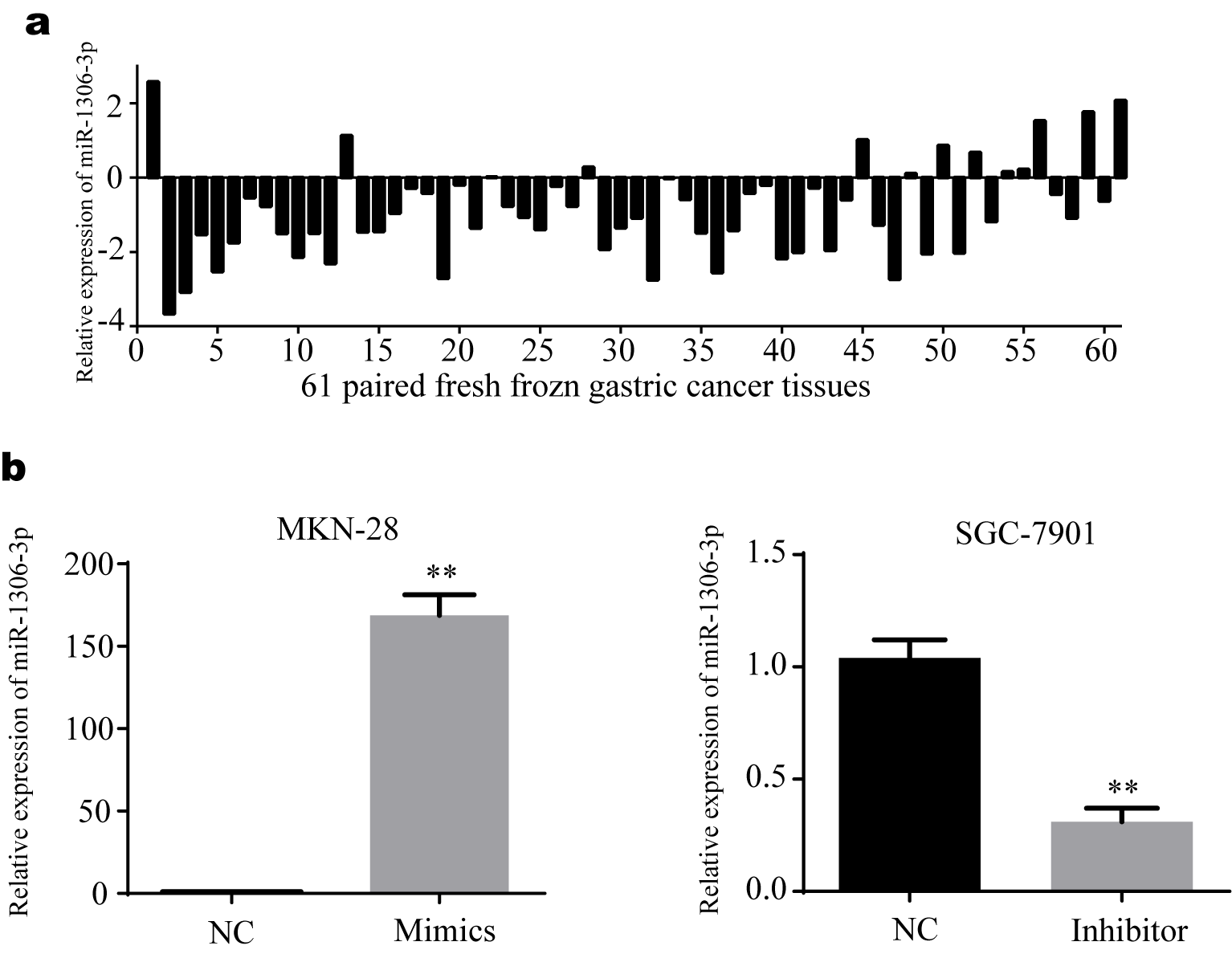

Supplement: Supplementary file 6 — Figure S5. The expression of miR-1306-3p. a The level of miR-1306-3p was down-regulated in 80.33% (49/61) gastric cancer tissues. b The efficiencies of transfection with mimics and inhibitor of miR-1306-3p were determined in MKN-28 and SGC-7901 cells. (TIF 272 kb) [file 12943_2019_1054_MOESM6_ESM.tif]

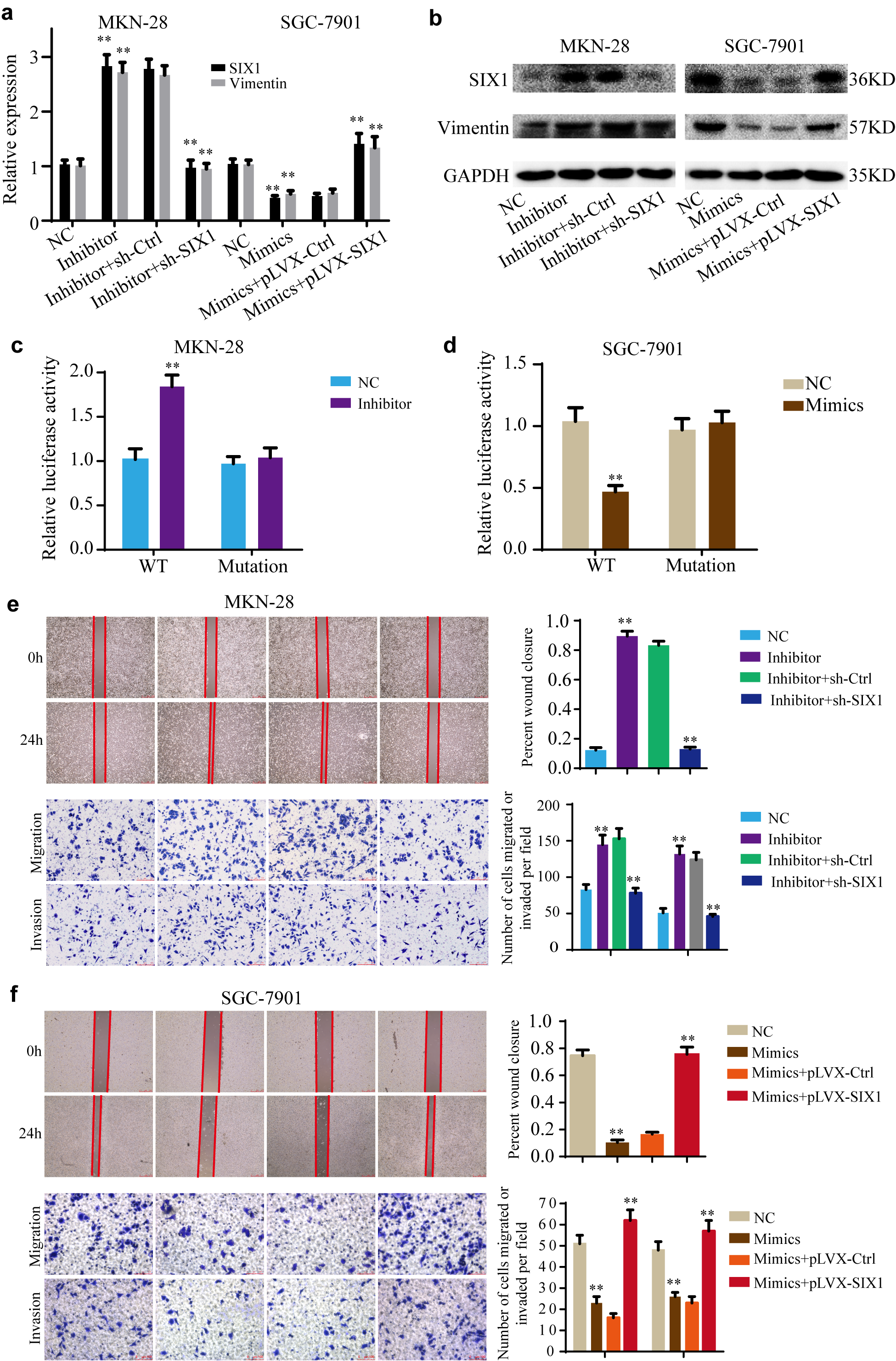

Supplement: Supplementary file 7 — Figure S6. MiR-1306-3p suppresses gastric cancer progression through directly targeting SIX1. a and b The effects of miR-1306-3p and SIX1 on the mRNA and protein expressions of SIX1 and Vimentin were detected by qRT-PCR (a) and western blotting (b). c and d The effects of inhibitor (c) and mimics (d) of miR-1306-3p on the luciferase activities of wild type of SIX1 mRNA 3′-UTR (WT) and mutant SIX1 mRNA 3′-UTR (Mutant) were detected in MKN-28 and SGC-7901 cells. e and f The effects of miR-1306-3p and SIX1 on the mobility, migration and invasion were detected by wound healing and transwell assays in MKN-28 and SGC-7901 cells. All data are presented as the mean ± SEM of three experiments. **p < 0.01. (TIF 5078 kb) [file 12943_2019_1054_MOESM7_ESM.tif]
